# Research on rockburst prevention systems based on the attenuation law of coal and rock vibration wave energy

**DOI:** 10.1038/s41598-024-57258-w

**Published:** 2024-03-18

**Authors:** Hai Rong, Zijian Wang, Petr Konicek, Liting Pan, Guoshui Tang, Vlastimil Kajzar, Yadi Wang

**Affiliations:** 1 College of Mining, Liaoning Technical University, Fuxin, 123000 China; 2 Department of Geomechanics and Mining Research, Institute of Geonics of the Czech Academy of Sciences, Ostrava, 70800 Czech Republic; 3 Chouzhou Polytechnic, Chuzhou, 239000 China

**Keywords:** Energy attenuation, Heterogeneous medium, Rockburst, Numerical simulation, Analytic hierarchy process, Energy science and technology, Engineering, Mineralogy, Petrology

## Abstract

During the coal and rock mass fracture process, elastic properties are released and vibration waves are radiated outward. The energy attenuation characteristics of these waves can describe the cumulative damage and elastic energy accumulation of the mass. To investigate coal and rock mass failure characteristics and energy attenuation rules during rockburst, numerical simulation and laboratory testing were utilized to study the energy transfer laws under various parameters. Six variables, including elastic modulus, Poisson’s ratio, bulk density, cohesion, internal friction angle, and void ratio, were selected to simulate the rockburst energy release process under different parameter combinations by adding surface pressure to the model. The coal and rock mass energy attenuation coefficient was obtained by fitting the node energy straight line using the least squares method. The six variables’ influence on vibration wave energy transfer was obtained using analytic hierarchy process program written in MATLAB, and a comprehensive calculation formula was proposed. Using the energy attenuation coefficient, the rock layer energy diffusion distance was calculated and compared with the roof collapse rock layer step distance, resulting in the roof rock layer cutting distance determination. By roof rock strata precutting, rockburst occurrence can be prevented, ensuring safe and efficient coal mine production.

## Introduction

Rockburst is a complex, dynamic disaster in coal mines. It is characterized by the sudden release of elastic energy in a violent and forceful manner, resulting in instantaneous destruction of coal and rock strata. This can cause coal and rock masses to be thrown and displaced, generating loud noise and airwaves and causing severe damage to property and people^1–3^. The deformation and destruction of coal and rock are essentially dynamic dissipation processes of coal and rock mass energy. To understand the coal rock fragmentation mechanism, it is important to analyze the energy dissipation that occurs after the instability of the coal rock dynamic system. Studying the failure mechanism and energy transfer law of coal and rock masses under dynamic loads is crucial in determining the energy dissipation law of coal and rock masses and its controlling effect on rockbursts. This knowledge is essential for preventing dynamic disasters in coal mines^4–9^.

Numerous scholars have extensively researched the rockbursts occurrence mechanism, the laws governing energy transfer and dissipation in coal and rock masses, and methods for preventing roof cutting and erosion. Through the results of their research, they have achieved significant and noteworthy results. Jiang^10,11^, for instance, developed the theory of the coal and rock mass impact effect, which incorporates the principles of energy transfer and conservation, and the analysis of rock mass properties, organizational failure, and weakening. By employing this theory, Jiang comprehensively analyzed and discussed the rockbursts formation mechanism using different cognitive methods. He has further revealed and discussed the ideas regarding rockburst and the technical strategies for prediction equipment research and development.

Building upon the geo-dynamic division theory, Zhang et al. identified the position of coalfield geo-dynamic conditions in controlling dynamic coal mine disasters by dividing the mining area structure and conducting dynamic evaluations. They have introduced the new perspective that "different mining areas, coal seams, and structures exhibit different laws governing dynamic disasters in coal mines," effectively guiding research, development, and practice of mine dynamic disaster prediction and prevention technology. Zhang and his team have also established a regional dynamic disaster prediction method based on geo-dynamic division, allowing for unit probability prediction of dynamic disasters in coal mines^12,13^. In addition, Ma et al.^14^ proposed rockbursts energy characteristics and mechanism elements and established an evaluation index and system for roadway rockburst mechanisms. They used the Analytic Hierarchy Process (AHP) to calculate the weight of each criterion layer and proposed a calculation method and classification standard for the comprehensive evaluation of results. Qi et al.^15^ studied rockburst behavior characteristics during mining of two working mine faces and combined it with temporal and spatial evolution analysis and microseismic energy event characteristics. By conducting numerical simulations of the tectonic stress distribution law in the overlying rock between mines, they obtained the rockburst generation mechanism during the mining of adjacent working faces between two adjacent mines under the condition of large faults and a thick gravel roof. Similarly, Cao et al.^16,17^ analyzed the post-mining energy evolution law and rockburst mechanism through theoretical analysis, numerical simulation, and field measurement to address the problem of frequent strong mine earthquakes in the high-stress area of a large-dip thick coal seam during the mining process of the working face. They formulated a prevention and control scheme for energy release and load reduction of directional deep drilling blasting of the roof plate based on energy distribution characteristics and the induced impact mechanism in the process of mining thick seams with a large dip angle. This scheme effectively reduces the inclined suspended roof stress and prevents rockburst occurrence in the high-stress area.

Song et al.^18^ have focused from an energy perspective on coal-rock system failure and collapse monitoring. By utilizing an energy-consuming Electromagnetic Radiation (EMR) monitoring system and the geophysical EMR method, they studied the coal rock failure with an impact tendency from the point of dissipation and discussed uniaxial compression dissipation energy characteristics and its main influencing factors.

Similarly, He et al.^19^ studied the failure and energy dissipation characteristics of coal-rock composite models. They conducted sample tests with different rock-coal strength ratios (RCSRs) and found that RCSRs have a significant energy consumption and mechanical property impacts of the samples. Research in their study also analyzed the energy mechanism and showed that the energy dissipation coefficient curve has a peak at the yield strength point as the RCSRs increase.

Furthermore, Xu et al.^20^ revealed the rock failure process through theoretical analysis of energy transfer during rock failure. By conducting numerical experiments based on the finite element method, their research studied the strain energy released by the testing machine under different loading system stiffness (LSS) loading conditions. The modeling results have shown that the strain energy released by the testing machine alone can greatly affect the rock failure process without increasing the energy supply. The research results can explain unstable rockburst failure in the test and the mechanism of some delayed rockbursts at a certain time after hole excavation.

Liu et al.^21^ proposed a rockburst risk prediction method by describing the coal and rock mass cumulative damage and elastic energy accumulation based on the stress adjustment and fracture outward radiation vibration waves energy characteristics. They also studied the energy attenuation law of microseismic vibration waves. Jiang et al.^22^ analyzed the relationship between rockbursts and mine shock and proposed three mechanical models: unstable rockburst of material, sliding stagger rockburst and unstable rockburst of structure. Zhang et al.^23^ determined the damage variable based on the surrounding rock energy dissipation rate ratio and the work done by the external force on the system. They concluded that the value of the fracture zone damage is greater than that in the plastic and elastic stress rise zones. Corresponding to the energy dissipation, they concluded that the energy dissipation in the fracture zone is greater than that in the plastic and elastic stress rise zones. To study the coal sample energy dissipation characteristics under dynamic load, Liu et al.^24^ conducted uniaxial impact compression tests on coal samples at different strain rates using the D50 mm SHPB test device at speeds ranging from 5.55 to 9.42 m/s. They analyzed coal sample energy and dissipation characteristics and found that the dissipation energy and energy consumption rate have a quadratic function relationship with the strain rate. With increasing strain rate, the dissipation energy increases significantly, while the energy consumption rate has the opposite relationship. These research results provide important references for understanding the occurrence process of coal and rock dynamic disasters such as rockburst.

Yang^25^ applied transmitted trough wave energy attenuation imaging technology to detect coal seam thickness changes. By analyzing the correlation between the energy attenuation coefficient and the coal thickness exposed by the roadway cut, he obtained the energy attenuation coefficient corresponding to the coal seam scouring boundary and coal thickness, achieving ideal results. Jin et al.^26^ calculated the dissipation energy according to cyclic loading and defined the material damage variable from the energy dissipation perspective, and they also studied the damage propagation law of relevant test results. Sun^27^ used fracture damage theory to analyze changes in the fracture collapse distance of the roof rock beam and the initial weighting strength of the fully mechanized top coal caving face after roof cutting and pressure relief. Gao et al.^28^ proposed using the temporary “roof cutting support plus portal support plus single pillar,” roadway support technology, the “W steel strip plus NPR constant resistance anchor cable,” wall control technology in the solid coal wall, and connected U-shaped steel in the gravel wall for gangue retaining support. Yang et al.^29^ compared and analyzed the test blasting effect under three different charge structures and obtained the optimal blasting crack rate. He et al.^30^ proposed a new gob-side entry retaining technology with roof cutting and pressure relief and deduced the roadway support resistance calculation method. Seinov and Chevkin^31^ showed the presence of open fissures in a solid rock mass markedly alters the fragmentation quality. Nateghi et al.^32^ introduced dynamic waves and their influence on underground structures, controlling methods of dynamic waves induced by blasting, and the design of surge tank storage shaft excavations in Gotvand dam, Iran, neighboring concrete structures in these underground storages. Singh et al.^33^ described the effect of blast-produced ground vibration on the damage potential to residential structures to determine safe levels of ground vibration for residential structures and other buildings in mining areas.

To improve rockburst control in mining areas, various rock control technologies have been proposed, such as shaped charge presplitting blasting, constant resistance and large deformation anchor cables, and dense single pillars near roadways. However, few studies have explored the relationship between rock control and the energy attenuation coefficient. Therefore, it is necessary to connect these two aspects. In addition, it is difficult to simulate the rockburst occurrence process. In the research conducted for this paper, a coal mass is selected as the research object, and the surface pressure is applied to simulate the dynamic breaking and energy dissipation process when rockburst occurs. The coal mass mechanical parameters are controlled to study the energy and structural characteristics after a rockburst. The influence and weight of six mechanical parameters, such as Poisson’s ratio, on the rockburst energy attenuation are obtained using the least squares method. Furthermore, the attenuation law of energy after rockburst is calculated using the MATLAB analytic hierarchy process. This energy attenuation law is then applied to the study of rockburst mine roof cutting measures and proposed for use in rockburst prediction and prevention in other mining areas based on the energy change.

## Numerical simulation of midas GTS NX blasting

### Numerical simulation of blasting

#### Model establishment and grid optimization

**Figure 1:**
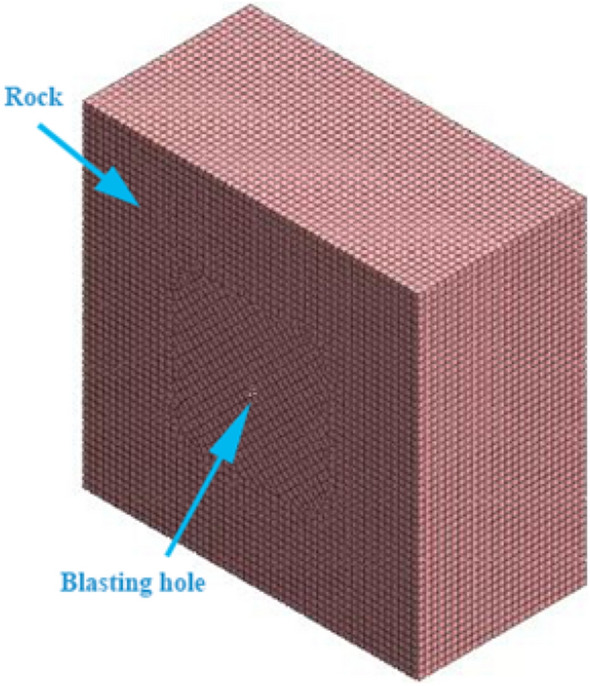
Spherical cavity mesh division.

To study the propagation law of blasting energy in coal rock, a model was established according to the actual situation. The model parameters include a cube with a side length of 20 m and a spherical cavity with a center radius of 0.2 m. To improve the accuracy, nodes are generated according to the principle of unified layout. A 0.3 m mixed grid is divided on the cube model edge, and the computer processing capacity and running speed are considered while ensuring accuracy. The grid center is divided into a mixed grid of 0.2 m. The model is shown in Fig. 1.

#### Selection of geometric parameters

After the model is established, the material parameters and model properties need to be defined and set. According to previous related research, for this paper, the elastic modulus, Poisson’s ratio, bulk density, cohesion, friction angle, and void ratio were selected as the main research object^34–40^. In this simulation, a basic model was first determined, and real model parameters were compared with the basic model parameters. This group of parameters is the default software common simulation data and is set as the basic model parameters. The Mohr Coulomb model with an elastic modulus of 25 GPa, Poisson’s ratio of 0.25, bulk density of 20 N/m^3^, cohesion of 4 MPa, friction angle of 30°, and void ratio of 0.25 was used as the basic model. This model is a common rock mass model. Different physical and mechanical parameters can be changed through the control variable method to investigate various parameter influence on the simulation results.

The Table 1 values demonstrate that in the analysis of the six physical and mechanical parameters that impact the coal and rock mass energy transmission, each mechanical parameter is evaluated using six different values. To simulate the energy attenuation law during the blasting process, application of the control variable method is applied to the blasting load. The obtained simulation results are then compared and analyzed to determine various mechanical parameter influence on energy attenuation.

**Table 1:** Blasting simulation mechanical parameters values.

Elastic modulus (GPa)	Poisson’s ratio	Bulk density (N m^−3^)	Cohesion (MPa)	Friction angle (°)	Void ratio
25	0.20	10	1	20	0.25
50	0.25	15	2	30	0.5
75	0.30	17	4	35	0.75
100	0.35	20	6	40	1
125	0.40	25	8	50	1.25
150	0.45	30	10	60	1.5

#### Boundary conditions setting

**Figure 2:**
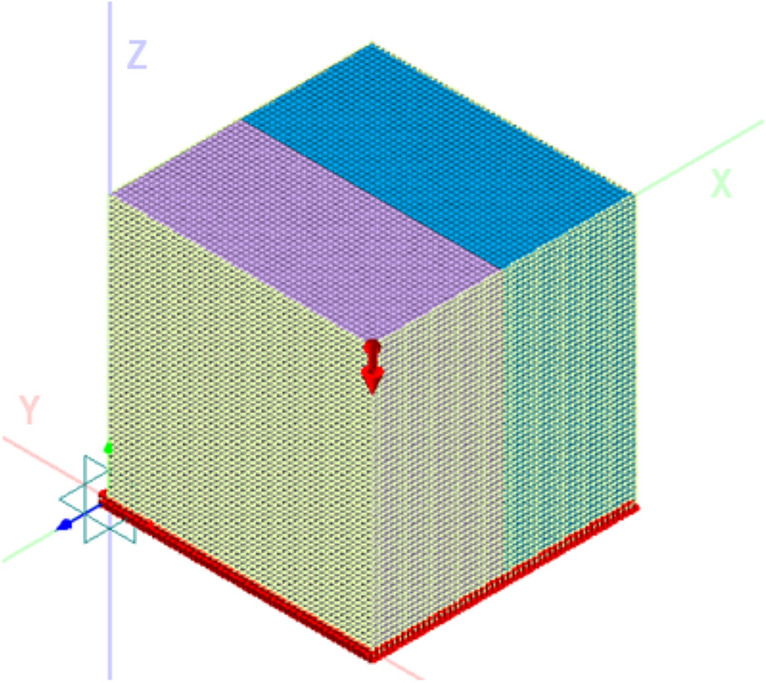
Diagram of blasting model boundary condition application.

During the Midas GTS finite element simulation, the boundary is divided into two categories: external and internal. The external boundary represents the soil, while the internal boundary represents the structural unit, the nodes between the structures, or the structural constraints. The external boundary is based on the actual site contact relationship, while the internal boundary is determined by the model’s appropriate size. For this study, the model is set to be isotropic, with a viscous damping boundary and a ground surface spring established in the unit settings. An eigenvalue solution analysis is carried out to consider the weight, as shown in Fig. 2. The study focuses on six key research objects: elastic modulus, Poisson’s ratio, bulk density, cohesion, internal friction angle, and void ratio. The influence of these six parameters on blasting energy attenuation is analyzed by setting each mechanical parameter to six different values. The blasting load is applied using the control variable method, and the energy attenuation law during the blasting process is simulated, as presented in Table 1.

#### Principle of blasting dynamic load

In this article, the author analyzed foreign research data and combined it with the actual engineering situation. The blasting load was calculated one by one using the formulas in Midas GTS software, and the maximum value among all methods was used as the experimental analysis load. The empirical formula proposed by the National Highway Research Institute of the United States organization was ultimately determined through calculation.

1 $$ P_{D} = 4.18 \times 10^{ - 7} \times S \times V^{2} /(1 + 0.8S) $$

In the Formula (1), PD is the blasting pressure, V is the blasting speed, and S is the explosive density.

#### Blasting load application

**Figure 3:**
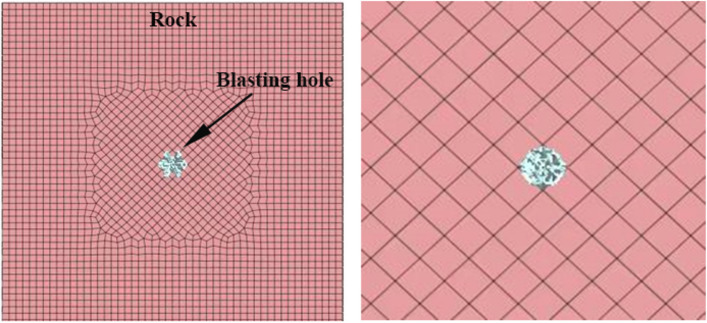
Enlarged view of model applied load.

Loads can be classified as earth pressure, water pressure, structural load, temperature load, etc. For this study, the Midas GTS blasting load is applied to the unit node inside the coal and rock mass model. This is illustrated in Fig. 3.

### Analysis of simulation results

**Figure 4:**
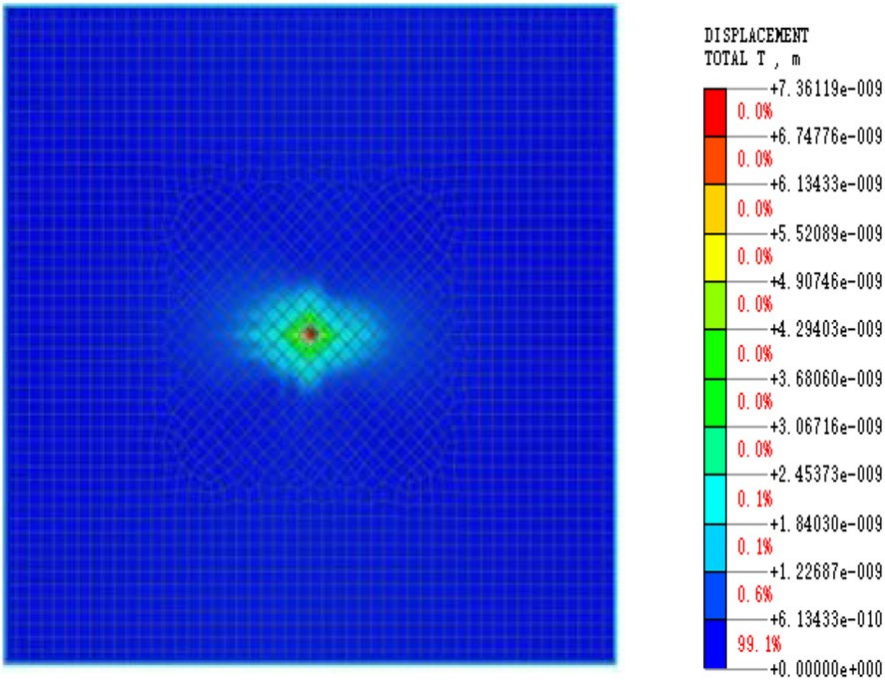
Cloud diagram of square model blasting.

**Figure 5:**
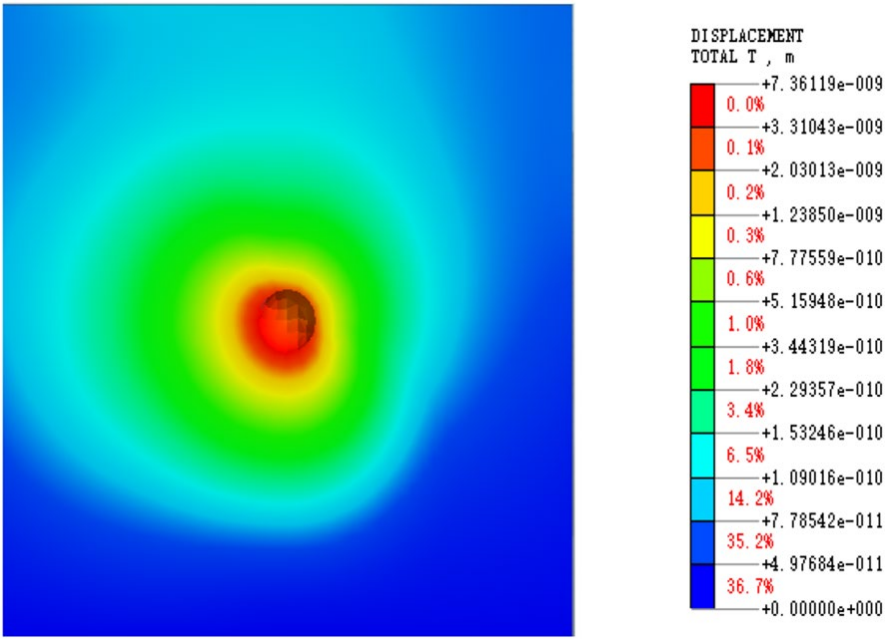
Cloud diagram of blasting hole.

In this paper, the Mohr‒Coulomb constitutive model was chosen for the elastoplastic constitutive model. Due to small cracks present in coal rock and soil, which results in strong inhomogeneity, the interaction between small cracks can cause them to merge and expand. Therefore, a homogeneous model was established for simulation, which makes it easier to determine the law of energy dissipation and attenuation after blasting occurs. The energy value was obtained by integrating the displacement and stress changes using the least squares method. Figure 4 shows the cube model blasting load cloud diagram, while Fig. 5 depicts the simulated blasting hole enlarged cloud diagram after the solution was obtained.

**Figure 6:**
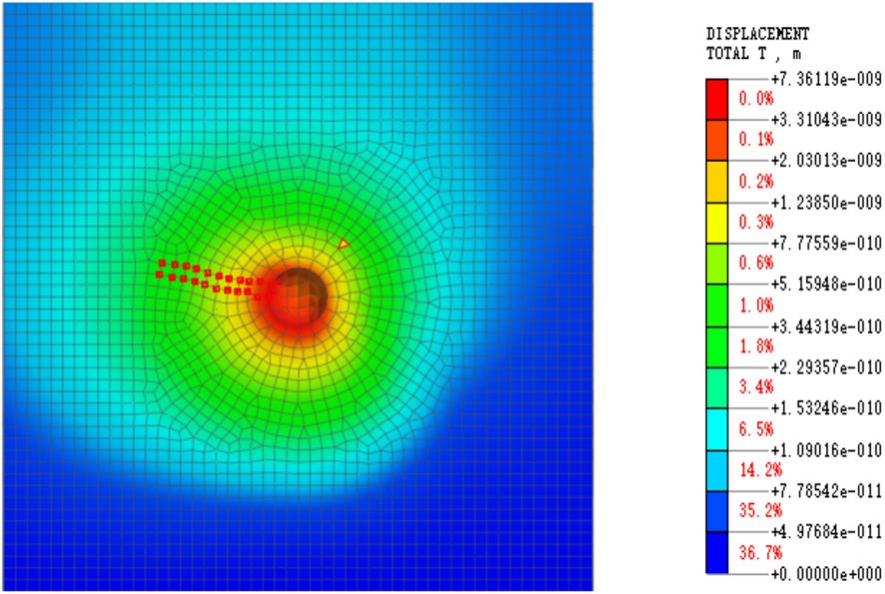
Displacement result extraction unit.

**Figure 7:**
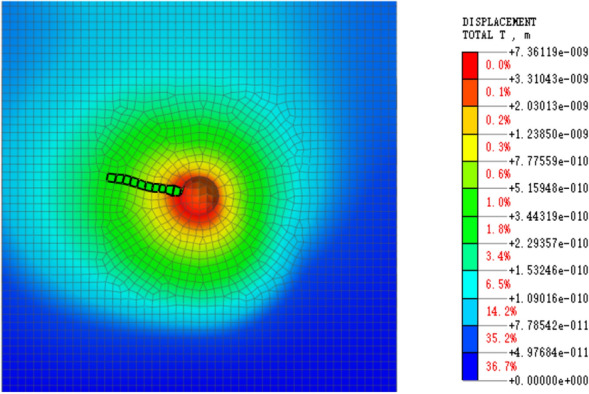
Stress result extraction unit.

The model’s eigenvalue is solved, and the linear time history is analyzed by integrating displacement and stress to determine the blasting simulation energy attenuation law. After the software simulation is complete, the displacement and stress data must be extracted. The displacement extraction scheme is depicted in Fig. 6, while the stress extraction scheme is shown in Fig. 7, which displays the stress change cloud diagram after blasting. To simplify the stress change law calculation, the stress change curve in the same direction and at the same distance is extracted from the image and marked with green cells.

**Figure 8:**
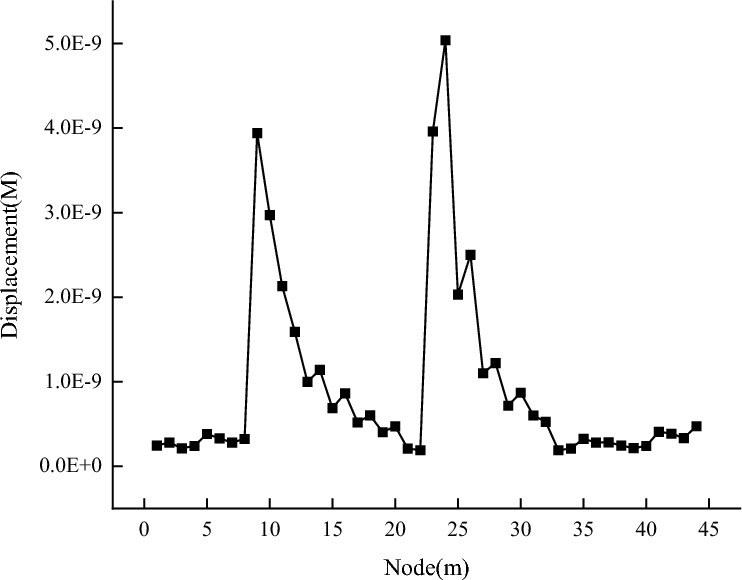
Displacement change curve.

**Figure 9:**
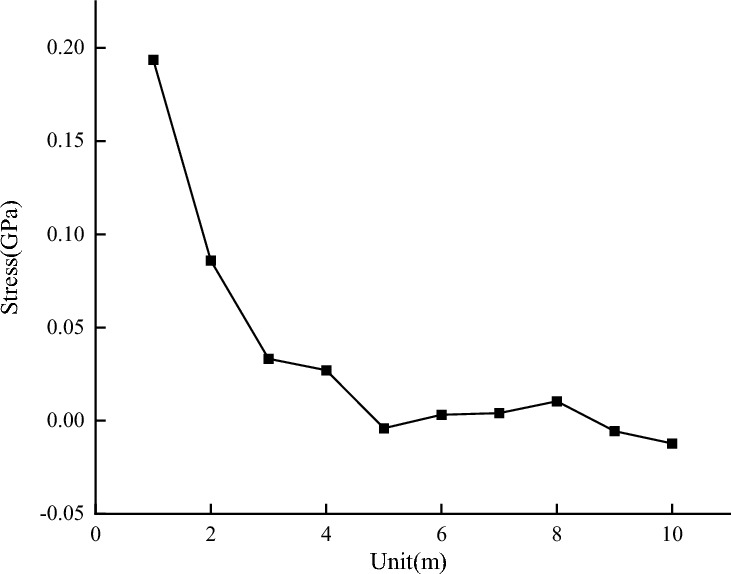
Stress change curve.

After running the simulation, stress and displacement data were extracted from the model and analyzed. Blasting generates many vibration waves in the coal mass. As the model node number increases, the vibration waves and their range also increase. The coal mass displacement value changes periodically in the waveform. It increases rapidly to the first peak, slowly decreases to the trough, then increases to the second peak, and slowly decreases again. This pattern is shown in Fig. 8. The stress change is relatively mild. When blasting occurs, an internal force is released instantly in the coal mass, causing the stress to reach its maximum value. As the number of module units increases, the stress decreases to a certain value and then gradually decreases, showing an exponential function distribution. The stress change curve is shown in Fig. 9. The compressive stress acting on the coal and rock mass causes compressive failure of the coal and rock mass. As a result, the compressive stress becomes zero, and the tensile stress gradually increases.

The cloud map displacement and stress data were extracted and plotted to observe their change trend. The energy change image was obtained through the integration of displacement and stress. The stress and displacement curves were fitted to obtain the energy change curve. This curve reveals the basic model energy attenuation law and coefficient.

**Figure 10:**
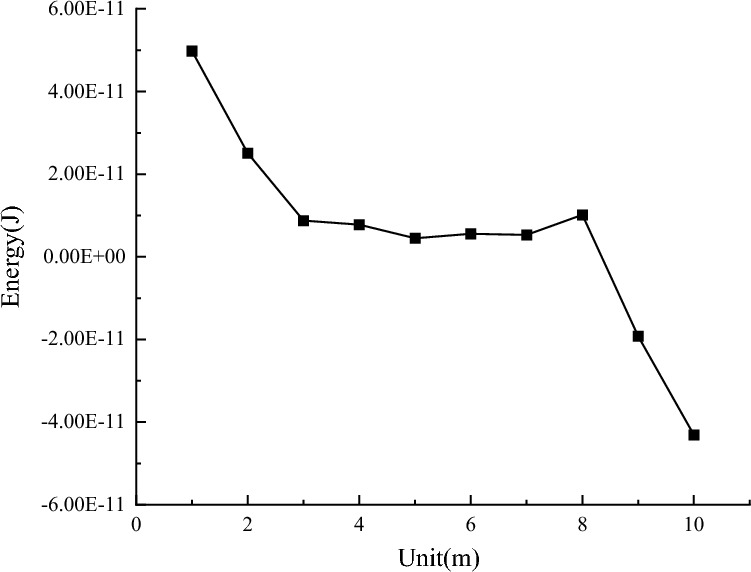
Energy change curve.

When the dynamic load is applied to the basic model, the coal and rock mass rupture, and the energy is rapidly released and propagates outward. The change image is shown in Fig. 10.

### Analysis of the mechanical parameter attenuation index

Through wave front geometric diffusion and medium damping effects, the peak velocity and particle energy in the vibration wave propagation process can be reduced. To understand the impact of different physical parameters on wave energy attenuation, we conducted a single-variable analysis and generated a node energy straight line using a minimum of two multiplications. The line slope, which is determined using the least square method, represents the change rate of the curve and the degree of attenuation of the image curve. This slope is referred to as the attenuation coefficient, denoted as K. We fitted each parameter, and the corresponding results are presented in Table 2.

**Table 2:** Corresponding arrangement coefficients of different physical parameters.

Elastic modulus (GPa)		Poisson’s ratio		Void ratio		Friction angle		Bulk density (kN m^−3^)		Cohesion (MPa)	
Parameter	k1	Parameter	k2	Parameter	k3	Parameter	k4	Parameter	k5	Parameter	k6
25	0.002	0.2	0.00201	0.25	0.00094	20	0.00194	10	0.0017	1000	0.002
50	0.00175	0.25	0.00212	0.5	0.002	30	0.00198	15	0.00174	2000	0.00213
75	0.00126	0.3	0.00223	0.75	0.00222	35	0.002	17	0.0019	4000	0.00227
100	0.00122	0.35	0.00261	1	0.00227	40	0.00203	20	0.0023	6000	0.00232
125	0.00121	0.4	0.00275	1.25	0.00268	50	0.00228	25	0.00277	8000	0.00251
150	0.00099	0.45	0.00371	1.5	0.00316	60	0.00234	30	0.00302	10,000	0.00268

**Figure 11:**
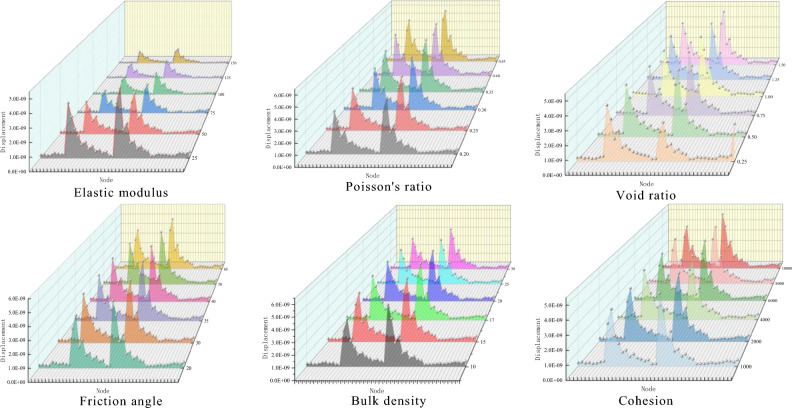
Different mechanical parameters effect on the energy transmission.

The blasting simulation was carried out by changing different mechanical parameters using the control variable method. Each parameter’s displacement change and stress change curves were obtained and analyzed. The energy change curve was obtained by integrating the stress and displacement data. As shown in Fig. 11.

#### Different mechanical parameter influence on displacement

The displacement change is inversely proportional to the change in elastic modulus, friction angle, bulk density, and cohesion and directly proportional to the change in void ratio and Poisson’s ratio. When the mechanical parameters are unchanged, the displacement changes show two peaks, and the displacement decays rapidly from the peak value to the lowest point in an exponential function. Each change trend is consistent, which is similar to periodic change.

#### Different Mechanical Parameters Influence on Stress Analysis

**Figure 12:**
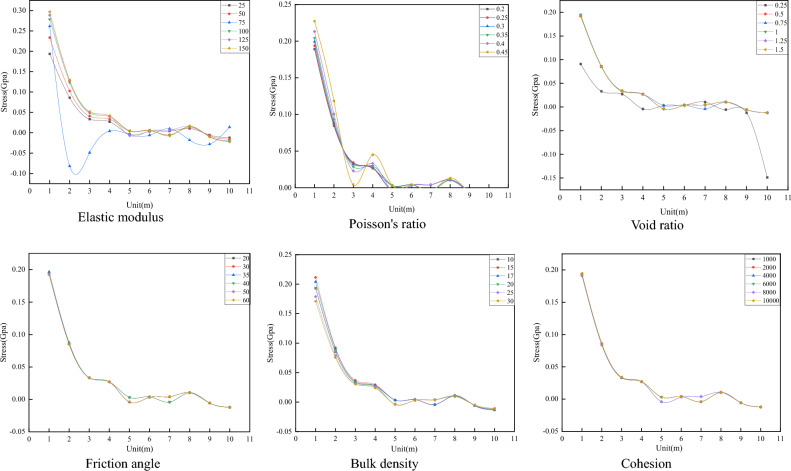
Different mechanical parameters effect on stress.

By extracting and processing stress data, changes in different mechanical parameters influence on stress is obtained. Positive values represent compressive stress, and negative values represent tensile stress, as shown in Fig. 12. The stress change is directly proportional to the change in elastic modulus, Poisson’s ratio, and cohesion and inversely proportional to the change in void ratio, friction angle, and bulk density. When the mechanical parameters are unchanged, with an increasing node displacement, the compressive stress gradually decreases to zero, and then the tensile stress increases rapidly.

#### Different mechanical parameters influence on energy variation

**Figure 13:**
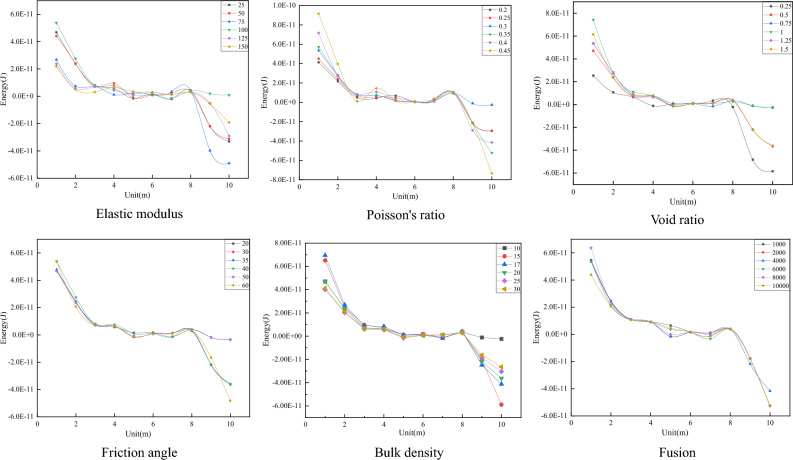
Different mechanical parameters influence on energy change.

The energy change data are extracted and analyzed to obtain the energy change trend under the influence of different mechanical parameters, as shown in Fig. 13. The results show that changes in Poisson’s ratio, void ratio, and friction angle are directly proportional to the energy change, while changes in elastic modulus and bulk density are inversely proportional to the energy change. When the coal and rock mass mechanical parameters remain unchanged, the energy generated by the work done by the compressive stress decreases gradually with the increase in model nodes and elements and finally decreases to zero. Then, the energy generated by the work done by the tensile stress increases gradually.

From simulation result observations, the elastic modulus has the greatest influence on the peak displacement value. With an increasing elastic modulus, the peak displacement value changes significantly. When the void ratio is 0.25, the third period appears first. Poisson’s ratio has the greatest influence on the peak displacement value, and regardless of its value, the peak displacement value is always the largest. Cohesion has the least influence on displacement, and the displacement change image data are basically coincident.

The peak stress corresponding to Poisson’s ratio is the largest, and the elastic modulus and void ratio have the greatest impact on the change amplitude of stress. When the elastic modulus is 75 GPa, the compressive stress is 0.27 GPa, the tensile stress is 0.1 GPa, and the change is 0.37 GPa. When the void ratio is 0.25, the compressive stress is 0.09 GPa, the tensile stress is 0.15 GPa, and the change is 0.24 GPa. Changes in friction angle, bulk density, and cohesion have little effect on stress, and the stress variation trends of the three mechanical parameters are basically the same.

The Poisson’s ratio and void ratio have the greatest influence on energy. When Poisson’s ratio is 0.45, the initial compressive stress work is 9 × 10^–11^ J, the tensile stress work is 7.5 × 10^–11^ J, and changes to 16 × 10^–11^ J. When the void ratio is 1.5, the initial compressive stress work is 6 × 10^–11^ J, the tensile stress work is 4 × 10^–11^ J, and changes to 10 × 10^–11^ J. Cohesion changes have energy little effect, and the energy change curve remains basically the same.

To sum up, Poisson’s ratio has the greatest impact on energy attenuation, followed by elastic modulus, void ratio, unit weight, internal friction angle and cohesion. The magnitude of the impact of each parameter is now clear, but its specific degree of impact and relative proportion are still difficult to be determined. Therefore, it is particularly important to determine the weight of the influence of various mechanical parameters on energy attenuation.

### Weight analysis using AHP

#### Establishing hierarchical structure

**Figure 14:**
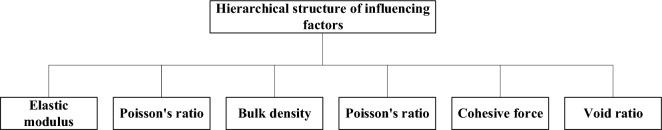
Hierarchical structure of influencing factors.

AHP is typically comprised of six steps: constructing a hierarchical structure, creating a judgment matrix, performing a consistency test, ranking the single level, ranking the total level, and making decisions. As the multilevel comprehensive evaluation model of the factors influencing the energy attenuation law of coal and rock masses in this simulation focuses solely on the mechanical parameters that impact energy attenuation and does not yet consider later schemes, the hierarchical structure solely consists of the target and index layers, as illustrated in Fig. 14.

#### Creating a judgment matrix

Taking into account the impact range of each parameter’s modification on the attenuation coefficient and the knowledge distilled from experts, the physical and mechanical parameters of coal and rock mass, such as elastic modulus, Poisson’s ratio, bulk density, and cohesion, are compared with one another, and ratios are established. Six mechanical parameters numerical values are presented in Table 3, revealing that the factors influencing energy attenuation of coal and rock mass have the following order of magnitude: Poisson’s ratio > elastic modulus > void ratio > bulk density > friction angle > cohesion.

**Table 3:** Quantitative values of physical and mechanical parameters.

	Poisson’s ratio	Elastic modulus	Void ratio	Bulk density (kN m^−3^)	Friction angle (°)	Cohesion (MPa)
Poisson’s ratio	1	2	3	5	6	7
Elastic modulus	1/2	1	2	4	5	6
Void ratio	1/3	1/2	1	3	4	5
Bulk density	1/5	1/4	1/3	1	3	4
Friction angle	1/6	1/5	1/4	1/3	1	3
Cohesion	1/7	1/6	1/5	1/4	1/3	1

#### Consistency test of the judgment matrix

① The consistency index (CI) is computed.
  2 $$ CI = \frac{{\lambda_{\max } - {\text{n}}}}{{{\text{n}} - 1}} $$
  where CI is the consistency index of the matrix, λ_max_ is the eigenvalue of matrix A, and N is the order of the matrix.
  The value of λ_max_ is set to 6.32, the value of n is 6, and the value of CI is 0.064 by substituting into Formula (2).
② The average random consistency index (CR) is calculated using RI, which is the mean random consistency index of the same order.
  3 $$ CR = \frac{CI}{{RI}} = \frac{0.064}{{1.26}} = 0.0508 $$
  where RI is the mean random consistency index of the same order.

When CR ≤ 0.1, the established judgment matrix’s consistency is deemed acceptable, indicating that the weight selection complies with the requirements. As the matrix in this step is of order 6, the RI is 1.26, which is substituted into Formula (3), and the CR value is 0.0508. As CR < 0.1, the matrix’s consistency adheres to the requirements.

**Figure 15:**
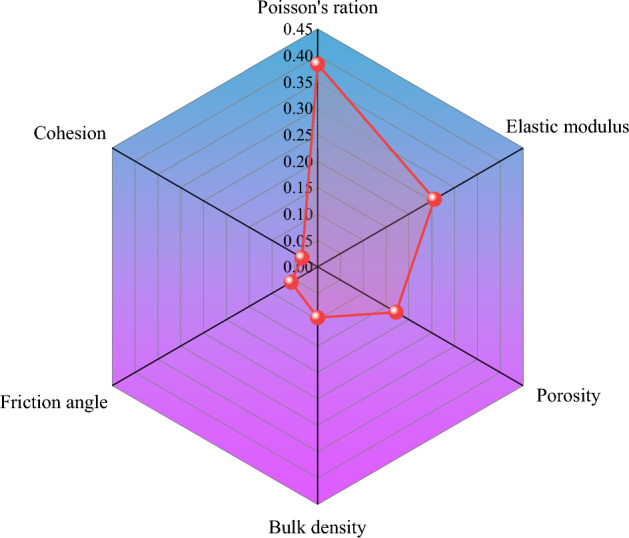
Physical and mechanical parameters weight comparison.

Judgment matrix A is constructed with reference to Table 4, and calculations are conducted using the weight calculation program coded in MATLAB. The results reveal that Poisson’s ratio weight of coal and rock mass is 0.3838, the elastic modulus weight is 0.2558, the void ratio weight is 0.1719, the bulk density weight is 0.0963, the friction angle weight is 0.0580, and the cohesion weight is 0.0342, as depicted in Fig. 15.

**Table 4:** Energy values corresponding to different falling heights of 300 g ball.

Falling height/cm	Energy/J (S1)	Energy/J (S2)	Energy/J (S3)	Energy/J (S4)
50	0.48	0.58	0.18	0.12
80	0.65	0.75	0.25	0.15
100	0.79	0.85	0.37	0.16

By totaling the attenuation coefficients across various value ranges of distinct physical coefficients, along with each physical coefficient influence weights, ultimately computing the weighted average of each coefficient, the energy propagation attenuation coefficient in the heterogeneous model can be derived. The formula is expressed as follows:

4 $$ K = \frac{{\sum\limits_{{{\text{i}} = 1}}^{6} {K_{{\text{i}}} {\text{k}}_{{\text{i}}} {\text{q}}_{{\text{i}}} } }}{{\sum\limits_{{{\text{i}} = 1}}^{6} {{\text{q}}_{{\text{i}}} } }} $$

where K is the coal and rock mass attenuation coefficient. q is the weight of the mechanical parameters and is 1, k is the multiple of the model parameters and the basic model parameters, and i is the serial number of the mechanical parameters affecting the energy attenuation. The specific calculation is as follows:

5 $$ \begin{aligned} K & = \frac{{K_{1} k_{1} \times 0.2558 + K_{2} k_{2} \times 0.3838 + K_{3} k_{3} \times 0.1719 + K_{4} k_{4} \times 0.0580 + K_{5} k_{5} \times 0.0963 + K_{6} k_{6} \times 0.0342}}{0.3838 + 0.2558 + 0.1719 + 0.0963 + 0.0580 + 0.0342} \\ K & = \frac{{2.07 \times 10^{ - 6} k_{1} + 2.61 \times 10^{ - 5} k_{2} + 3.06 \times 10^{ - 4} k_{3} + 5.22 \times 10^{ - 7} k_{4} + 7.03 \times 10^{ - 6} k_{5} + 2.77 \times 10^{ - 6} k_{6} }}{0.3838 + 0.2558 + 0.1719 + 0.0963 + 0.0580 + 0.0342} \\ \end{aligned} $$

The above has calculated the energy attenuation coefficient K. Then, according to the relevant research^17,41^, the rock mass medium discontinuity hinders vibration wave stress transmission and propagation of energy. In the process of vibration wave propagation, there is an internal relationship between particle peak velocity and vibration wave energy. Peak velocity and energy have a negative exponential relationship with propagation distance. Then, according to the calculation method of blasting vibration wave energy at the measuring point, as shown in Formula (6), the vibration wave energy attenuation law caused by blasting is analyzed and verified.

6 $$ E_{{\text{r}}} = E_{0} {\text{e}}^{{ - K{\text{r}}}} $$

where *E*_*0*_ is the vibration energy at the source (J) and *E*_*r*_ is the vibration energy at the measuring point. r is the distance between the measuring point and the source (m).

## Coal and rock mass energy attenuation law verification

### Experimental setup

#### Energy dissipation test

**Figure 16:**
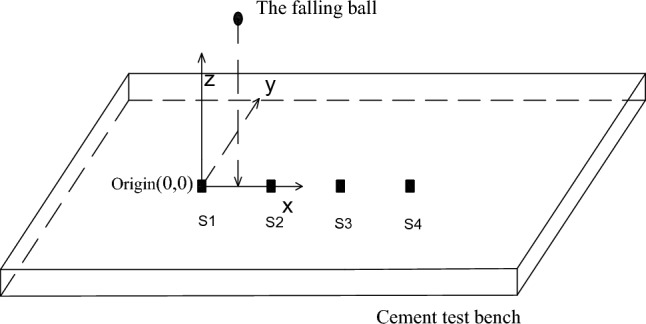
Ball falling test three-dimensional schematic diagram.

**Figure 17:**
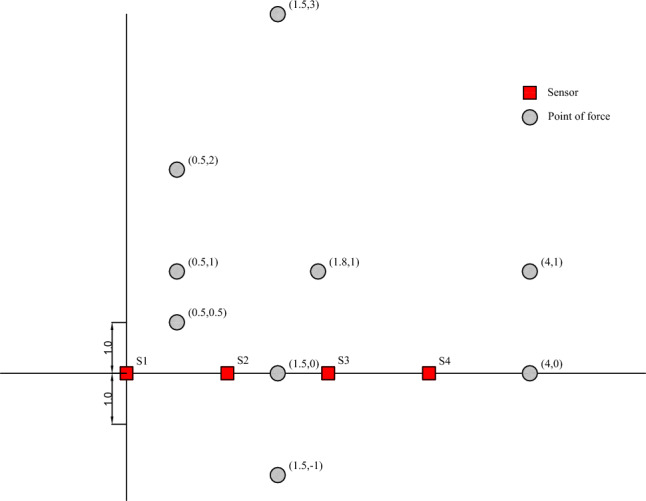
Sensor and ball falling force point position diagram.

**Figure 18:**
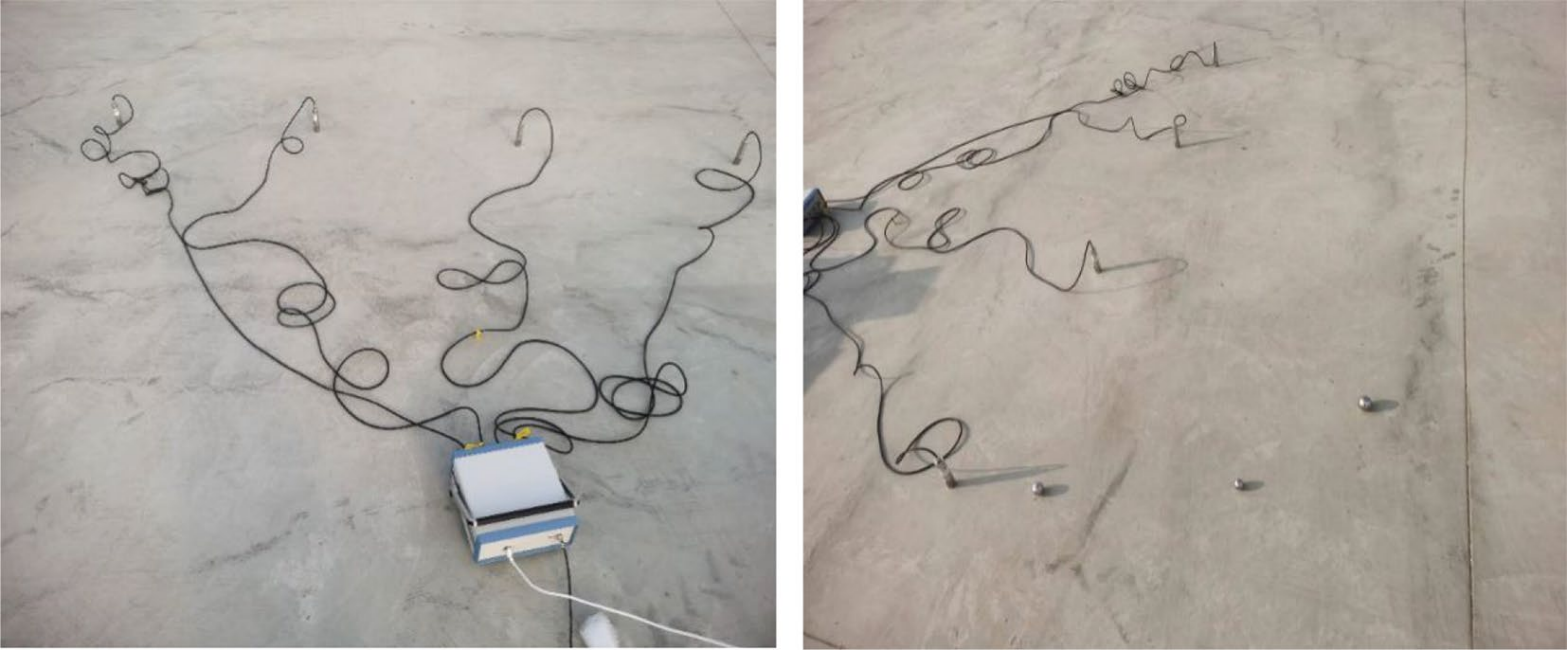
Diagram of sensor monitoring horizontal ground energy dissipation experiment.

During rockburst or blasting events, coal and rock mass instantaneously release a considerable amount of energy, which dissipates to the surroundings following a certain law. To analyze the energy attenuation law, it is essential to examine the energy value in the same direction, in the same horizontal plane, and at different distances from the blasting source. Four sensors, namely, S1, S2, S3, and S4, are arranged side by side on the concrete test bench at 1 m intervals. The position of the first sensor, S1, is considered the origin (0, 0), and the focus points of small steel ball landing are placed at points (0.5, 1), (0.5, 2), (1.5, 0), (1.5, − 1), (4, 1), (4, 0), etc. The schematic diagrams are shown in Figs. 16 and 17. The falling ball test is carried out at different sensor position distance points, and the test is repeated multiple times. The energy attenuation law and attenuation coefficient are analyzed by studying the trend, peak value, and area of the data image. The test process is depicted in Fig. 18.

#### Simulated source testing process

**Figure 19:**
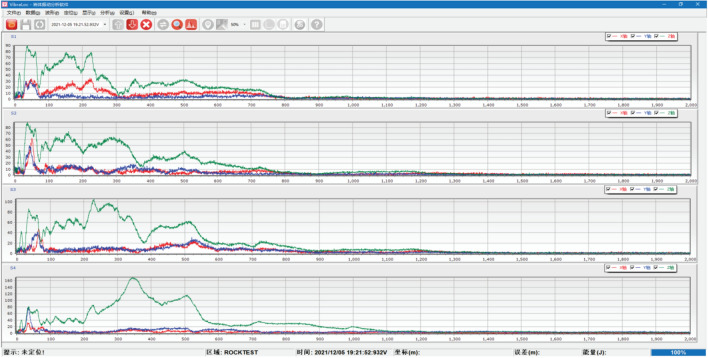
Sensor spectrum acquisition display.

During the simulated source testing, different impact forces are generated on the top of the test bench as the steel ball falls, resulting in energy generation. The energy signal is transmitted from the sensor to the vibration demodulator and then to the computer. The data are processed to generate a curve image, as shown in Fig. 19.

In the testing process, four sensors are placed side by side on the concrete test bench, with a 1 m interval. Sensor S1 is located at the origin (0, 0), and the other three sensors are distributed along the horizontal axis, with coordinates S2 (1, 0), S3 (2, 0), and S4 (3, 0). The closer the sensor is to the force point, the greater the influence of gravity on the steel ball, resulting in greater energy being obtained. The change curve of the shock wave and energy is obtained by controlling the variable method to change the uniform steel ball mass and the steel ball’s fall height.

#### Test data acquisition

**Figure 20:**
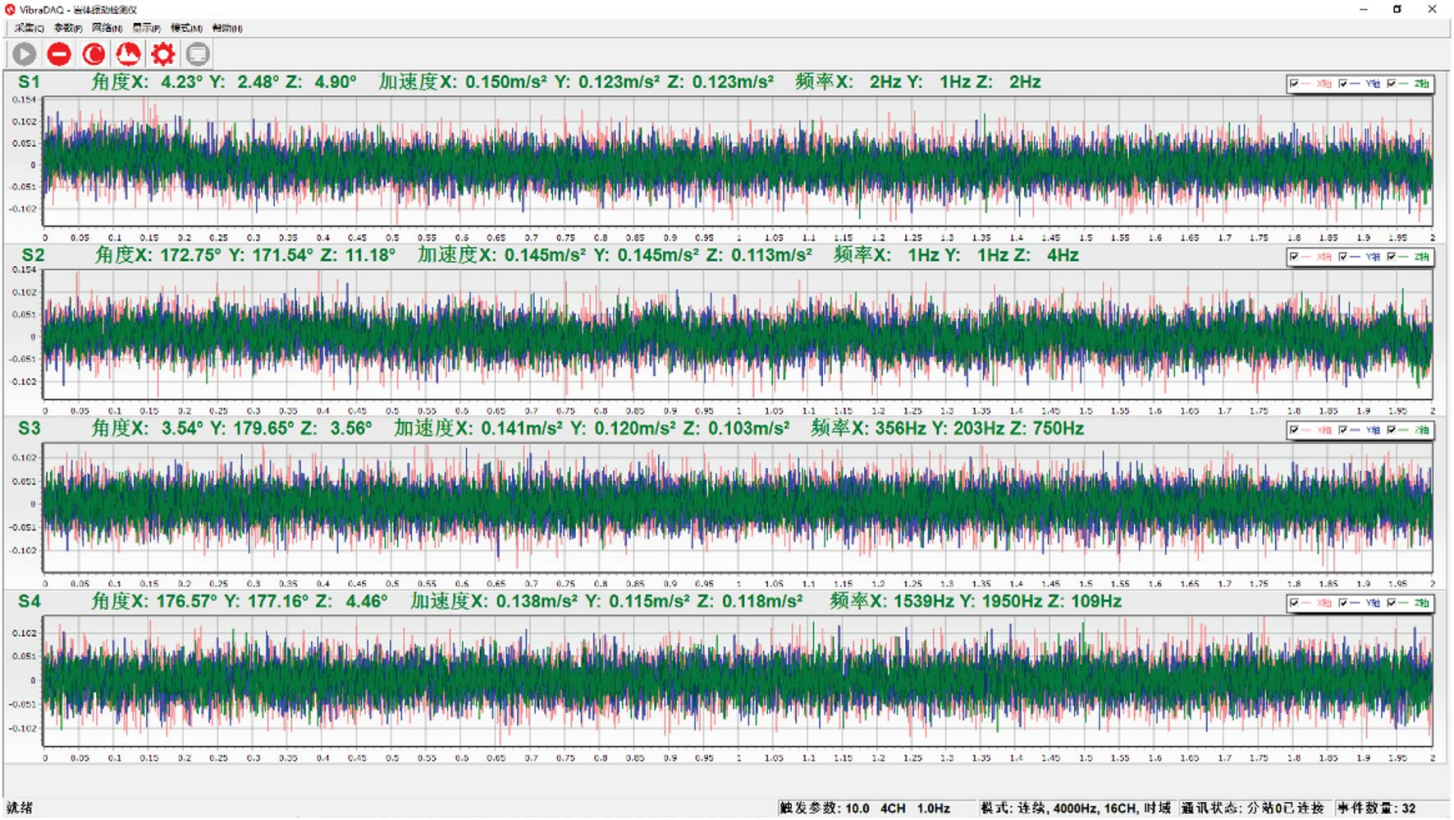
Waveform data display interface.

**Figure 21:**
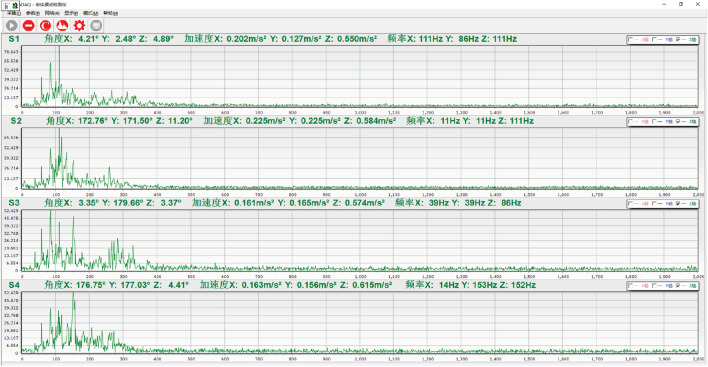
Frequency domain switching interface.

After data processing, the necessary image is obtained, which includes signal components in three directions (X, Y, and Z) and the signal’s amplitude and acceleration. To ensure data authenticity and accuracy, the tests were conducted in both quiet and noisy environments. The resulting data displayed are not filtered and are the raw data, as shown in Fig. 20. The spectrum display of the data is shown in Fig. 21.

### Data processing

#### Time–frequency conversion principle analysis

① Fourier transform.
  Fourier transform is the basis of signal processing. Before analysis, the original signal can be transformed from the time domain to the frequency domain through a Fourier transform. Let the signal f (t) satisfy the boundedness criterion in any interval in the infinite interval and satisfy Formula (7).
  7 $$ f(t) \in L^{2} (R),\int_{ - \infty }^{ + \infty } {|f(t)| < { + }\infty } $$
  The Fourier transform is defined as:
  8 $$ F(W) = \int_{ - \infty }^{ + \infty } {{\text{e}}^{ - jwt} f(t)} $$
  In the Fourier transform calculation process, the key point is to integrate f (t) on R. To make the obtained spectrum information more accurate, it is necessary to discretize the time domain signal and frequency domain signal. Discrete time series DFT {f (t)} is defined as:
  9 $$ X_{k} = F(f_{n} ) = \sum\limits_{n = 0}^{n - 1} {f_{n} e}^{{ - i\frac{2nk}{N}}} $$
② Power spectral density (PSDz)
  PSD amplitude represents the frequency band effective value square and includes the complex form, amplitude, and phase. The power spectral density describes the unit frequency energy distribution and can be used to accurately calculate the sensor energy. PSD expression is defined as:
  10 $$ S(w,\tau ) = \int_{R} {f(t)} g(w - \tau )e^{ - jwt} dt $$
  To comprehensively and systematically analyze the signal over its entire time domain, the selected window function g (t) should meet the basic compromise conditions. Therefore, the short-time Fourier transform (STFT) provides better results for stationary signals analysis.

#### Test data preprocessing analysis

**Figure 22:**
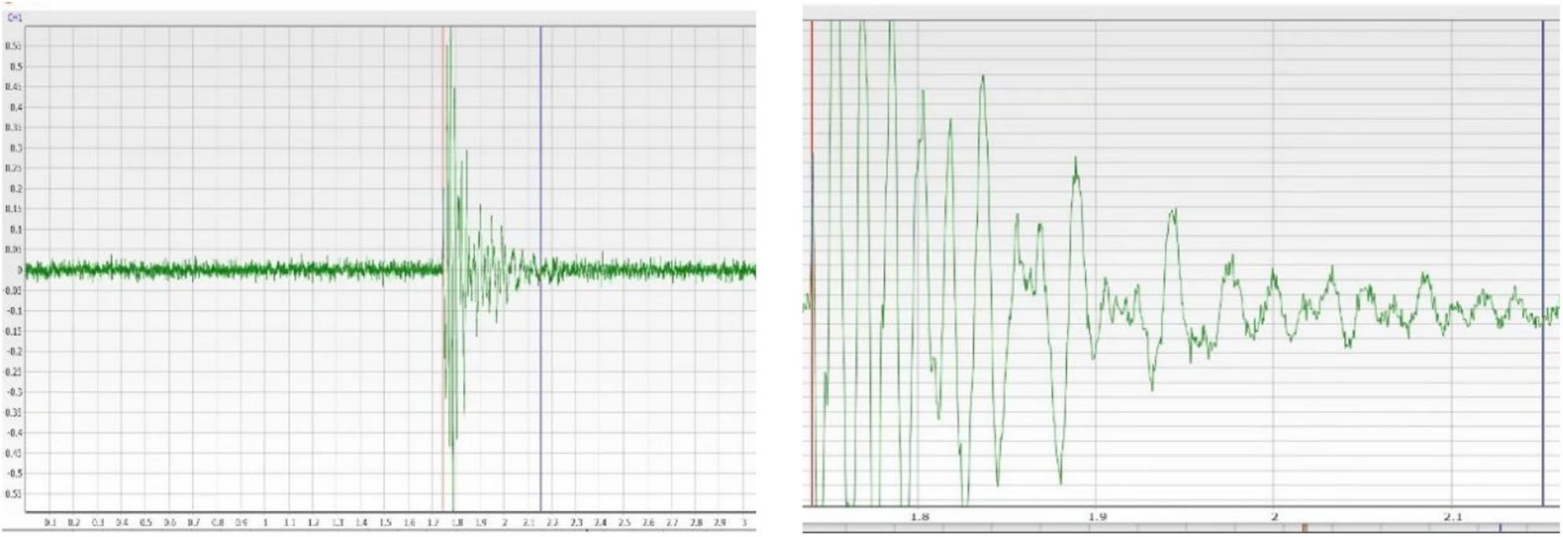
Enlarged view of waveform data.

During the test, the main force acting on the concrete test bench is the gravity of the steel ball, with negligible impact from other forces. The resulting vibration wave is collected by the sensor, and the data are generated and saved, as shown in Fig. 22.

**Figure 23:**
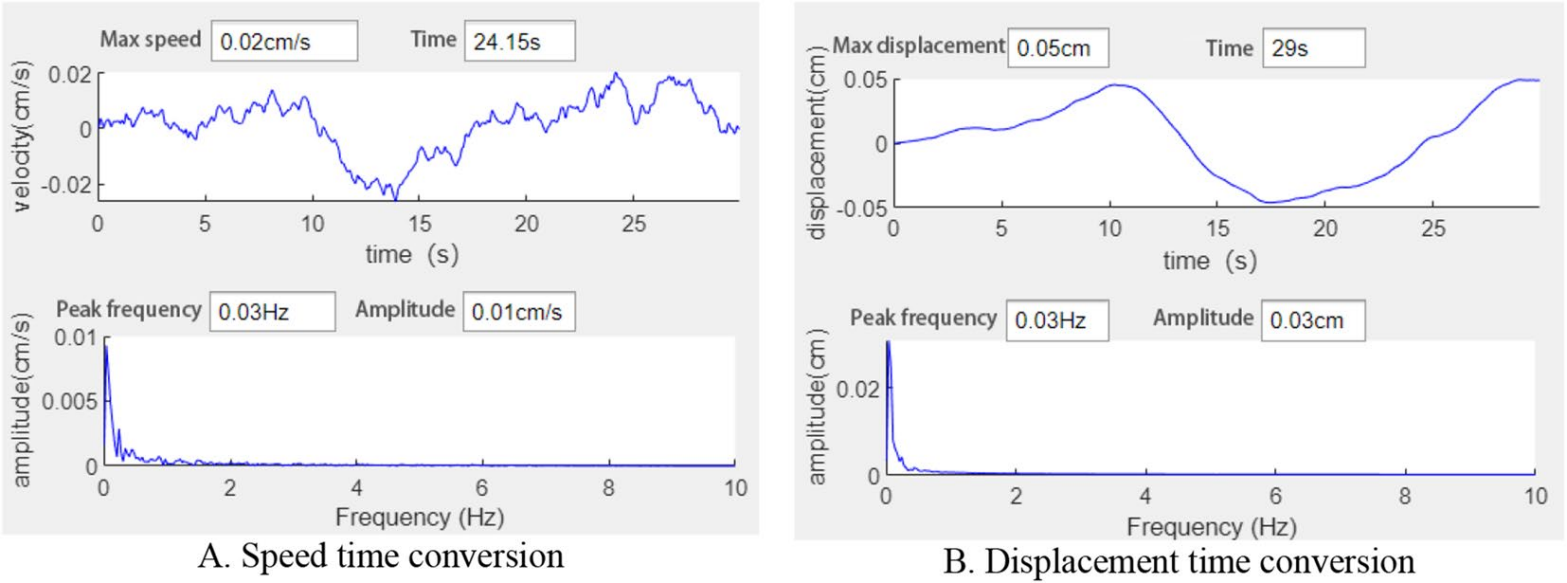
Time history conversion.

Because the vibration wave propagation velocity and amplitude are closely related to the medium through which they pass, the medium conditions largely determine the vibration wave propagation characteristics in this case. The original data are further processed to obtain both velocity and displacement time–frequency characteristics based on the acceleration time history, as shown in Fig. 23A,B.

PSD of a time signal f (t) represents how signal or time series power is distributed across different frequencies. When analyzing signal energy, PSD can be used to reflect the vibration energy in each frequency range. It can clearly show the main vibration amplitude and the regularity of the sinusoidal vibration.

### Result analysis

**Figure 24:**
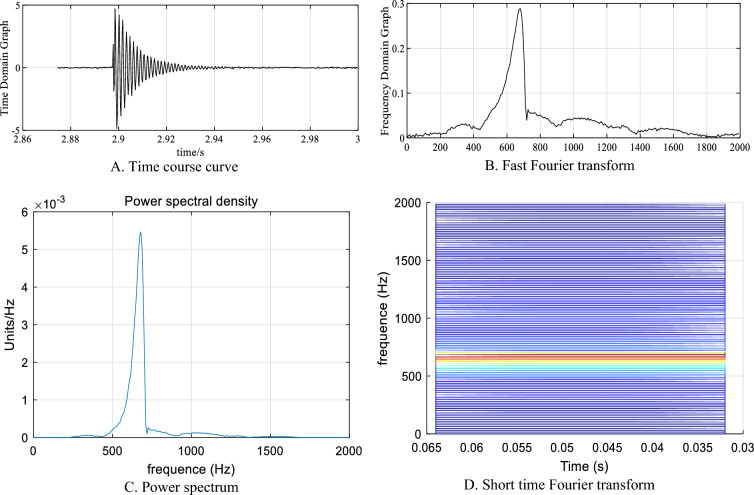
Time frequency conversion results.

Based on the above analysis and data, MATLAB was used to compile a time–frequency conversion program. After time history baseline adjustment results obtained from the test, 500 rows of data columns were obtained after filter processing. The original time history results can be seen in Fig. 24A. Using the control variable method, the time history curve was converted into frequency domain records using the time–frequency conversion method to obtain the amplitude and power spectrums, and the short-time Fourier transform results of the fast Fourier transform, as shown in Fig. 24B–D.

It is difficult to identify the dominant frequency components in vibration data through time history analysis alone. The spectrum curve obtained through the fast Fourier transform (FFT ) can clearly show the dominant frequency distribution with the largest amplitude. However, direct FFT of random signals truncates the signals into energy signals for processing. In physical terms, power spectral density represents the signal energy (in units of J) in a unit frequency. In the time domain, power is equal to work/time, while in the frequency domain, power is equal to work/frequency. The area under the power spectral density curve represents the total signal energy, which is the sum of the squares of all amplitudes, as shown in Formula (11).

11 $$ E = \mathop {\lim }\limits_{T \to \infty } \int_{ - T}^{T} {|f(t)} |^{2} dt $$

#### Variable focus

**Figure 25:**
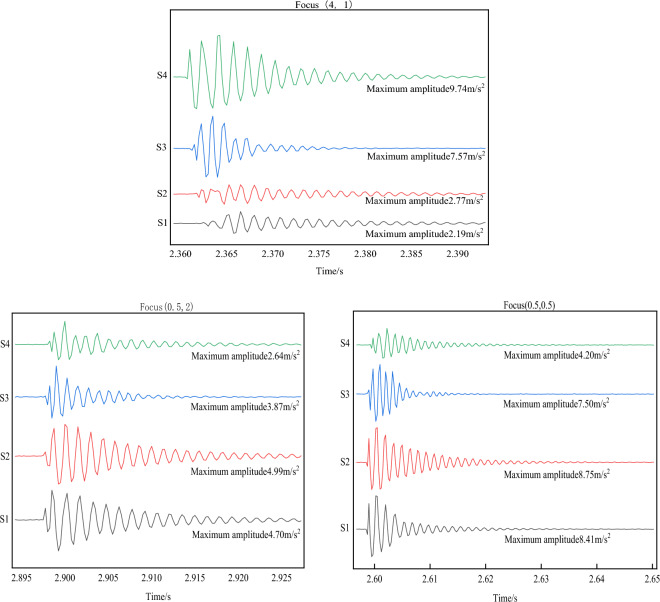
Amplitude variation curve of different sensors.

When a small ball with a mass of 300 g falls from a height of 50 cm at different points (4, 1), (0.5, 2), and (0.5, 0.5), S1, S2, S3, and S4 sensor amplitude changes are observed, as shown in Fig. 25.

① At point (4, 1), the S4 sensor is closest to the impact point, and the amplitude is 9.74 m/s^2^. The S1 sensor is the farthest from the impact point, and the amplitude is 2.19 m/s^2^.
② At point (0.5, 2), the distances between sensors S1 and S2 from the impact point are the same, and the amplitudes are 4.70 m/s^2^ and 4.99 m/s^2^, respectively. Sensor S4, which is farthest from the impact point, has an amplitude of 2.64 m/s^2^.
③ At point (0.5, 0.5), the distances between sensors S1 and S2 from the impact point are the same, and the amplitudes are 8.75 m/s^2^ and 8.41 m/s^2^, respectively. Sensor S4, which is farthest from the impact point, has an amplitude of 4.2 m/s^2^.

This indicates that when the sensor is at the same distance from the impact point, the energy received by the sensor is basically the same, while the energy received by the sensor farther from the impact point is lower.

#### Drop height as a variable

**Figure 26:**
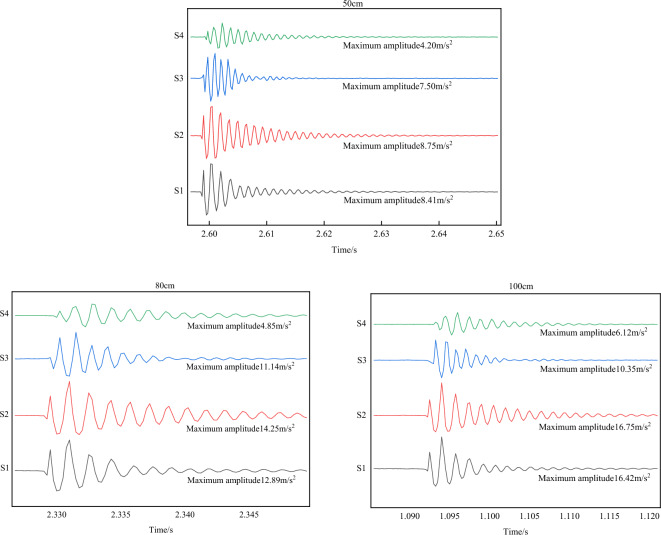
Different falling heights energy change diagram.

The experiment was conducted with a small ball of 300 g mass dropped from different heights (50 cm, 80 cm, and 100 cm) to point (0.5, 0.5) on the test bench. The four sensor energy change values were measured and plotted, shown in Fig. 26. Sensors S1 and S2 were equidistant from the force point, showing similar maximum value amplitudes. On the other hand, the sensor S4 amplitude, which was farthest from the impact point, was the minimum.

① When the ball was dropped from a height of 50 cm, sensor S1 and S2 amplitudes were 8.41 m/s^2^ and 8.75 m/s^2^, respectively. Sensor S3 and S4 amplitudes were 7.5 m/s^2^ and 4.2 m/s^2^, respectively.
② When the ball was dropped from a height of 80 cm, sensor S1 and S2 amplitudes were 12.89 m/s^2^ and 14.25 m/s^2^, respectively. Sensor S3 and S4 amplitudes were 11.14 m/s^2^ and 4.85 m/s^2^, respectively.
③ When the ball was dropped from a height of 100 cm, sensor S1 and S2 amplitudes were 16.42 m/s^2^ and 16.75 m/s^2^, respectively. Sensor S3 and S4 amplitudes were 10.35 m/s^2^ and 6.12 m/s^2^, respectively.

The results show that as the ball drop height increases, the initial impact point energy also increases. As the drop height decreases, energy gradually decreases, and the energy attenuation of the nearby sensors is fast, while that of the distant sensors is slow. Table 4 displays different falling height energy values.

#### Ball mass as a variable

**Figure 27:**
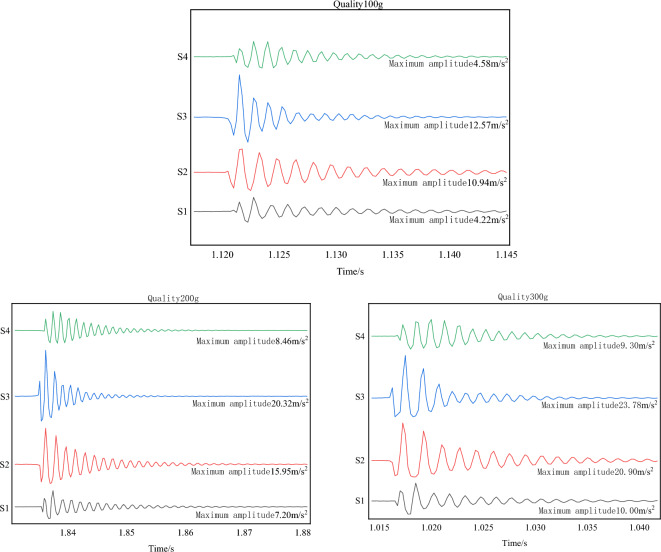
Different ball mass energy change diagram.

The experiment was carried out with small balls of masses 100 g, 200 g, and 300 g dropped vertically from a height of 100 cm to point (1.8, 1) on the test bench. The four sensor energy change values were measured, and the results are shown in Fig. 27. Sensor S3 was closest to the impact point, exhibiting the maximum amplitude peak, followed by sensors S2 and S4. Sensor S1, which was the farthest from the force point, showed the smallest amplitude. The energy of each sensor was calculated from the power spectral density of the Fourier transform, as presented in Table 5.

**Table 5:** Corresponding ball energy values with different weights falling from 100 cm.

Ball weight/g	Energy/J (S1)	Energy/J (S2)	Energy/J (S3)	Energy/J (S4)
100	0.74	1.91	1.95	0.83
200	1.35	2.43	2.67	1.41
300	1.62	3.31	3.35	1.73

① When the ball mass was 100 g, sensor S3 amplitude was 12.57 m/s^2^, sensor S2 amplitude was 10.94 m/s^2^, sensor S4 amplitude was 4.58 m/s^2^, and sensor S1 amplitude was 4.22 m/s^2^.

② When the ball mass was 200 g, sensor S3 amplitude was 20.32 m/s^2^, sensor S2 amplitude was 15.95 m/s^2^, sensor S4 amplitude was 8.46 m/s^2^, and sensor S1 amplitude was 7.2 m/s^2^.

③ When the ball mass was 300 g, sensor S3 amplitude was 23.78 m/s^2^, sensor S2 amplitude was 20.9 m/s^2^, sensor S4 amplitude was 11.3 m/s^2^, and sensor S1 amplitude was 9.00 m/s^2^.

After calculating the sensor energy, we can calculate the initial ball energy using its gravitational potential energy as it falls. If the ball has a mass of m and falls from a height of h, its gravitational potential energy is given by *mgh*. Thus, we can express the initial energy as *E*_0_ = *mgh* − *E*_p_, where *E*_p_ is the energy lost due to any other forms of friction or air resistance during the fall.

Once the ball lands, it possesses elastic potential energy, which we can measure during the second bounce test. This test shows that the elastic potential energy still makes up a considerable proportion of the ball's energy, despite some energy lost during the first bounce. The energy attenuation range is 26%, meaning that the energy has decreased by that amount compared to the initial energy. The remaining 74% of the energy is accounted for by vibration energy acting on the measuring point.

The initial vibration energy wave is 0.586 J after the calculation of the gravitational potential energy minus the elastic energy. According to Formula (6), the energy at the landing points of S1, S2, S3 and S4 is 0.48 J, 0.58 J, 0.18 J and 0.12 J, respectively, attenuation coefficients are 0.004, 0.0002, 0.008 and 0.006, respectively, and the attenuation coefficient is 0.00455 after the average value is obtained.

### Mechanical parameter test

#### Cement test block uniaxial compressive strength test results

Cement test block uniaxial compressive strength was tested individually, and the results are presented in Table 6.

**Table 6:** Cement test block compressive strength test results.

Serial No	Cross sectional area (mm)	Failure load (kN)	Compressive strength (MPa)	Average compressive strength (MPa)
1	2634.40	144.72	59.93	45.805
2	2558.33	126.9	49.60	
3	2588.54	120	46.36	
4	2648.36	122	46.07	
5	2577.52	96.6	37.48	
6	2558.13	90.54	35.39	

#### Cement test block elastic modulus and Poisson's ratio test results

The cement test block elastic modulus and Poisson's ratio were tested, and the test results are displayed in Table 7.

**Table 7:** Cement test block elastic module and Poisson’s ratio test results.

Serial No	Elastic modulus (GPa)	Poisson's ratio	Average elastic modulus (GPa)	Average Poisson’s ratio
1	35.163	0.27	34.912	0.255
2	33.15	0.24		
3	37.61	0.24		
4	39.65	0.26		
5	30.24	0.25		
6	33.592	0.27		

#### Cement test block internal friction angle and cohesion measurement results

The cement test block internal friction angle and cohesion were tested, and the test results are shown in Table 8.

**Table 8:** Cement test blocks internal friction angle and cohesion test results.

Lithology	Uniaxial compressive strength (MPa)	Friction angle (°)	Cohesion (MPa)
Test block	51.62	47.47	4.8

#### Table 9 summarizes the cement test block mechanical parameters

**Table 9:** Cement test block mechanical parameters.

Mechanical parameters	Elastic modulus (GPa)	Poisson's ratio	Friction angle(°)	Cohesion (MPa)	Bulk density (kN m^−3^)	Void ratio
numerical value	35	0.26	47.47	4.8	20	0.5

The cement test block mechanical parameters were obtained and compared with the basic model. Table 10 shows the ratios.

**Table 10:** Summary of proportional coefficients of mechanical parameters.

Mechanical parameters	Basic model	Cement	Scale factor
Elastic modulus/GPa	25	35	1.4 (k1)
Poisson's ratio	0.25	0.26	1.04 (k2)
Void ratio	0.5	0.5	1 (k3)
Friction angle/°	30	47.47	1.58 (k4)
Bulk density kN/m^3^	20	20	1 (k5)
Cohesion /MPa	4	4.8	1.2 (k6)

The proportional coefficient K in the table was used to calculate the rock mass vibration energy attenuation in Formula (4) of Chapter 2 of the paper. When substituted into the formula, the resulting energy attenuation coefficient K2 for the cement test block was 0.00607. This value is consistent with the attenuation coefficient of 0.00455 calculated in “Result analysis” section of this chapter, as they are both on the same order of magnitude.

## Field application

### Causes of roof cutting in thick rock strata of rock burst mines and discrimination of hard thick rock strata

#### Causes of roof cutting in thick strata of rock burst mines

According to previous study results, hard and thick rock roofs are the main rockburst force source^42^. Lan et al.,^43^ based on rock pressure behavior and microseisms data from a mining area located in Xinjiang, China, concluded that factors such as the impact of coal mining technology and the low support quality led to a coal body stress concentration and elastic energy accumulation, making the hard and thick roof the main source of rockburst. Based on this, they proposed the principle of timely cutting off of the hard roof and conducting pressure relief blasting on the coal body to prevent rockburst. In view of the rockburst disaster in the coal mining face under the condition of hard and thick roof in a mine, Li et al.^44^ adopted the measures of roof cutting blasting between supports in the dangerous rockburst period and achieved good results after testing, which effectively reduced the coal body stress in front of the working face. Through layer discrimination, the working face rock structure instability scale can be determined. Combined with the following description, it can better guide the development of mine rockburst prevention and control work, such as roof cutting and pressure relief. Aiming at the technical problem of impact and roadway deformation in a deep mining area of disturbance, Li et al.^45^ studied linear dense roof cutting anti-scour protection roadway technology, determined the best technical parameters through theoretical analysis and simulation research, and verified the effectiveness in practice. Taking the working face of the Nantun Coal Mine as the engineering background, Sun Xiaoming et al.^46^ studied the key parameters of gob-side entry retaining with roof cutting and pressure relief in a thin coal seam. Through the roof stress state analysis during the thin coal seam working face mining process, the method of effectively cutting off the goaf roof and the retaining roadway roof stress transfer is proposed. The research results have important reference significance for the popularization and application of roof cutting and pressure relief gob-side entry retaining technology in thin coal seam mining.

#### Hard rock strata structural characteristics analysis

After coal seam mining, the overlying strata are unstable. Because the overlying rock strata have hard and thick characteristics with a small expansion coefficient, the overlying strata movement range is wide. At the same time, there are often multiple hard rock strata above the coal seam, which are different according to their spatial location and control effect. According to the research results of Dr. Li Yunpeng's doctoral dissertation “Research on structural instability characteristics and hydraulic fracturing control technology of hard rock strata”^47^, hard rock strata are divided into low, medium and high hard rock strata. The progressive and composite instability plate structure system model of hard rock stratum, low cantilever structure → middle masonry structure → high pressure arch structure is established. Hard rock strata in different spatial positions have different structures. The spatial structure characteristics of hard rock strata are described as follows:

Through the analysis of the structural characteristics of hard rock strata, based on the key strata theory, the overlying strata are divided into low, medium and high key strata. The hard rock layers of each key layer form different structures, and the energy released by the instability of the hard rock structure will control different mine pressure behaviors.

① Low hard rock cantilever structure. Due to hard rock strata characteristics, the volume expansion coefficient is small after crushing, and there is always free space under the low hard rock strata. After the initial low hard rock strata instability, a cantilever structure is formed.
② Medium hard rock masonry structure. With the initial low hard rock strata instability and periodic instability, the overlying strata failure height develops to the middle hard rock strata. After the initial middle hard rock strata instability, the masonry structure is formed.
③ High hard rock pressure arch structure. With low hard rock and medium hard rock instabilities, the overlying strata failure height develops to the high hard rock position. The rock strata controlled by the high-level hard rock strata are unstable. The hard rock strata located in the upper part of the high-level hard rock strata bend and sink and contact the rock mass in the goaf. At this time, the hard rock strata cannot meet the instability conditions, and the hard rock strata above the high-level hard rock strata are no longer unstable. After the initial instability of the high-level hard rock stratum, the masonry structure can also be formed. With the high-level hard rock stratum as the boundary, the spatial structure formed has a large scale and affects the goaf behind the working face. It can be regarded as a ‘pressure arch’ structure with a certain thickness.

After coal seam mining, through analysis of the hard rock strata structural characteristics, the overlying hard rock strata can be divided into low hard rock strata, medium hard rock strata and high hard rock strata. The low hard rock stratum forms a ‘cantilever structure’, the middle hard rock stratum forms a ‘masonry structure’, and the high hard rock stratum forms a ‘pressure arch structure’. Different structural instabilities control different mine pressure effects.

#### Hard rock structures instability scale calculation method

① Initial instability scale calculation of low, medium and high hard rock strata
  After the coal seam is mined, the overlying strata will move and destabilize to a secondary equilibrium state. Through the analysis of the overlying strata structural characteristics in the stope, the low, medium and high hard strata that control the overlying strata movement and instability can be determined. When the working face advances to reach its ultimate instability scale, the first rock layer instability will occur.
  According to the small deflection theory of thin plates, the calculation methods of the initial instability scale of low, medium and high hard rock strata are the same. The solution is shown in Eq. (12).
  12 $$ \left\{ {\begin{array}{*{20}l} {b_{1i} = 4\sqrt {\frac{{3q_{i} a_{i}^{2} M_{si} + 8a_{i} M_{si} \sqrt {3q_{i} M_{si} } }}{{q_{i} \left( {3q_{i} a_{i}^{2} - 64M_{si} } \right)}}} } \hfill & {\left( {2x_{i} \le a_{i} } \right)} \hfill \\ {b_{1i} = \frac{{8\sqrt 3 a_{i}^{2} \sqrt {q_{i} M_{si} } }}{{3qa_{i}^{2} - 48M_{si} }}} \hfill & {\left( {2y_{i} \le b_{i} } \right)} \hfill \\ \end{array} } \right. $$
  where $x_{i} = \frac{{b_{i} \sqrt {3a_{i}^{2} + b_{i}^{2} } - b_{i}^{2} }}{{2a_{i} }}$, $y_{i} = \frac{{a_{i} \sqrt {a_{i}^{2} + 3b_{i}^{2} } - a_{i}^{2} }}{{2b_{i} }}$. Where *b*^1^_*i*_ is the first instability scale (m) of the *i*th hard rock layer; *a*_*i*_ is the hanging length (m) of the hard rock along the direction of the working face; and *q*_*i*_ is the load (MPa) of the first hard rock layer.
  *M*_*si*_ is the ultimate bending moment of the i-layer hard rock (N·m),
  $$ M_{si} = \frac{1}{6}\sigma_{si} h_{i}^{2} ; $$
  where *h*_*i*_ is layer i hard rock strata thickness (m) and *σ*_*si*_ is the tensile strength of layer i hard rock (MPa).
② Periodic low hard rock instability scale calculation.
  The initial low hard rock strata instability will lead to periodic instability as the working face continues to advance.
  Periodic low hard rock instability scale solution is shown in Eq. (13).
  13 $$ \left\{ {\begin{array}{*{20}l} {b_{2i} = \frac{{2\sqrt 6 a_{i}^{2} \sqrt {q_{i} M_{si} } }}{{3q_{i} a_{i}^{2} - 24M_{si} }}} \hfill & {\left( {y_{i} \le b_{i} } \right)} \hfill \\ {b_{2i} = \sqrt {\frac{{192M_{si}^{2} + 32\sqrt 3 \sqrt {M_{si}^{3} \left( {q_{i} a_{i}^{2} + 12M_{si} } \right) + 6q_{i} a_{i}^{2} M_{si} } }}{{q_{i} \left( {3q_{i} a_{i}^{2} - 64M_{si} } \right)}}} } \hfill & {\left( {2x_{i} \le a_{i} } \right)} \hfill \\ \end{array} } \right. $$
  where $$x_{i} = \frac{{b_{i} \sqrt {6a_{i}^{2} + 4b_{i}^{2} } - 2b_{i}^{2} }}{{2a_{i} }}$$, $$y_{i} = \frac{{a_{i} \sqrt {a_{i}^{2} + 12b_{i}^{2} } - a_{i}^{2} }}{{4b_{i} }}$$.
  where b^2^_i_ is the periodic low hard rock instability scale (m).
③ Periodic instability medium and high hard rock strata scale calculation.
  With the continuous working face advancement, the periodic low hard rock stratum instability will lead to the initial middle hard rock stratum instability scale. The calculation method is as follows. After the initial middle hard rock stratum instability, periodic instability will also occur. Similarly, the high hard rock stratum will also produce initial and periodic instabilities. According to the theory of small deflection of thin plates, the periodic medium and high hard rock strata instability scale calculation method is the same, as shown in Formula (14).
  14 $$ \left\{ {\begin{array}{*{20}l} {b_{3i} = \sqrt {\frac{{\left( {18 + 12\sqrt 2 } \right)q_{i} a_{i}^{2} M_{si}^{{}} + 16a_{i} M_{si} \sqrt {\left( {51 + 36\sqrt 2 } \right)q_{i} M_{si} } }}{{q_{i} \left( {3q_{i} a_{i}^{2} - 64M_{si} } \right)}}} } \hfill & {\left( {2x_{i} \le a_{i} } \right)} \hfill \\ {b_{3i} = \frac{{\sqrt {1190} a_{i}^{2} \left( {3q_{i} a_{i}^{2} + 34M_{si} } \right)}}{{210\left( {q_{i} a_{i}^{2} - 16M_{si} } \right)}}} \hfill & {\left( {y_{1i} + y_{2i} \le b_{i} } \right)} \hfill \\ \end{array} } \right. $$
  where $$x_{i} = \frac{{\left( {8\sqrt 2 - 12} \right)b_{i}^{2} + b_{i} \sqrt {\left( {9\sqrt 2 - 12} \right)a_{i}^{2} + \left( {136\sqrt 2 - 192} \right)b_{i}^{2} } }}{{a_{i} }}$$; $$y_{1i} = \frac{{a_{i}^{2} }}{{8b_{i} }}\left( {2k + 1} \right)\left( {\sqrt {1 + \frac{24k}{{\left( {1 + k} \right)\left( {1 + 2k} \right)}} \cdot \left( {\frac{{b_{i} }}{{a_{i} }}} \right)^{2} } - 1} \right)$$, *k* = 0.7; $$y_{2i} = \frac{{a_{i}^{2} }}{{8b_{i} }}\left( {2 + \frac{1}{k}} \right)\left( {\sqrt {1 + \frac{24k}{{\left( {1 + k} \right)\left( {1 + 2k} \right)}} \cdot \left( {\frac{{b_{i} }}{{a_{i} }}} \right)^{2} } - 1} \right)$$, *k* = 0.7.

In the formula, b^3^_i_ is the periodic medium–high hard rock strata instability scale (m).

According to the above calculation formula, the layers of low, medium and high hard rock strata and their respective instability scales can be calculated, and then the movement law of the working face advancing and the overlying strata instability can be determined.

The working face rock stratum structure instability scale is determined, and it is necessary to analyze the energy release law of hard rock stratum instability to guide mine rockburst prevention and control, such as roof cutting and pressure relief.

### 13,230 hard rock stratum structural characteristics and judgment

According to Dr. Li Yunpeng's method of identifying hard rock strata in rockburst mines, the layers of low, medium and high hard rock strata in the 13,230 working face of the Gengcun Coal Mine were calculated^47^.

The 13,230 working face of the Gengcun Coal Mine is located on the east side of the belt downhill of the East Third mining area, reaching the Gengcun and Qianqiu Coal Mine boundaries in the east, and the goaf of the 21,121 working face of the Qianqiu Coal Mine is adjacent to its mine boundary. In the north is the mined-out area of the 13,210 working face, and in the south is the untapped 2–3 coal entity. The working face dip length is 196 m, and the minable strike length is 971 m. The working face ground elevation is + 625 to + 647 m. The average working face mining depth is 633 m, and the maximum mining depth is 686 m.

The working face mining thickness is 13–38 m, the average thickness is 17.4 m, and the coal seam dip angle is 9°–13°. The working face adopts the strike longwall backward fully mechanized top coal caving mining method, and the roof is managed by the natural caving method, in which the coal cutting height is 2.6 m. Table 11 shows the 13,230 working face borehole column shape and mechanical parameters.

**Table 11:** Summary of the Gengcun Coal Mine 13,230 working face borehole columns and mechanical parameters.

Serial No	Rock stratum name	Thickness (m)	Volumetric force kN (m^3^)	Poisson’s ratio	Elastic modulus (GPa)	Tensile strength (MPa)
Y43	Siltstone	11.08	28.68	0.24	59.29	7.78
Y42	Conglomerate	9.05	27.97	0.21	37.10	6.42
Y41	Siltstone	1.95	28.68	0.24	59.29	7.78
Y40	Conglomerate	2.8	27.97	0.21	37.10	6.42
Y39	Siltstone	7.2	28.68	0.24	59.29	7.78
Y38	Conglomerate	2.4	27.97	0.21	37.10	6.42
Y37	Siltstone	7.2	28.68	0.24	59.29	7.78
Y36	Conglomerate	6.5	27.97	0.21	37.10	6.42
Y35	Siltstone	27.6	28.68	0.24	59.29	7.78
Y34	Glutenite	2.95	27.97	0.21	37.10	6.42
Y33	Conglomerate	7.35	27.97	0.21	37.10	6.42
Y32	Siltstone	1.2	28.68	0.24	59.29	7.78
Y31	Conglomerate	1.05	27.97	0.21	37.10	6.42
Y30	Siltstone	2.1	28.68	0.24	59.29	7.78
Y29	Conglomerate	1.04	27.97	0.21	37.10	6.42
Y28	Siltstone	11.1	28.68	0.24	59.29	7.78
Y27	Conglomerate	0.8	27.97	0.21	37.10	6.42
Y26	Siltstone	1.04	28.68	0.24	59.29	7.78
Y25	Conglomerate	0.6	27.97	0.21	37.10	6.42
Y24	Medium sandstone	0.75	26.97	0.24	59.29	7.78
Y23	Conglomerate	0.81	27.97	0.21	37.10	6.42
Y22	Siltstone	14.3	28.68	0.24	59.29	7.78
Y21	Conglomerate	1.04	27.97	0.21	37.10	6.42
Y20	Siltstone	9.40	28.68	0.24	59.29	7.78
Y19	Conglomerate	3.90	27.97	0.21	37.10	6.42
Y18	Siltstone	1.50	26.97	0.24	59.29	7.78
Y17	Conglomerate	7.20	27.97	0.21	37.10	6.42
Y16	Siltstone	10.05	28.68	0.24	59.29	7.78
Y15	Fine sandstone	6.85	28.68	0.24	59.29	7.78
Y14	Siltstone	7.15	28.68	0.24	59.29	7.78
Y13	Fine sandstone	4.35	28.68	0.24	59.29	7.78
Y12	Mudstone	1.30	26.97	0.24	32.31	4.67
Y11	Siltstone	2.05	26.97	0.24	59.29	7.78
Y10	Medium sandstone	1.30	28.68	0.24	59.29	7.78
Y9	Mudstone	1.48	26.97	0.24	32.31	4.67
Y8	Siltstone	1.87	28.68	0.24	59.29	7.78
Y7	Fine sandstone	4.00	28.68	0.24	59.29	7.78
Y6	Siltstone	2.92	28.68	0.24	59.29	7.78
Y5	Breccia	1.98	26.97	0.21	37.10	6.42
Y4	Fine sandstone	0.70	28.68	0.24	59.29	7.78
Y3	Mudstone	1.6	26.97	0.24	32.31	4.67
Y2	Carbonaceous mudstone	0.66	13.44	0.24	32.31	4.67
Y1	Mudstone	22.26	26.97	0.24	32.31	4.67
2–3# coal						

Table 12 shows the hard rock stratum structural characteristics.

**Table 12:** Gengcun coal mine working face 13,230 hard rock layer distribution.

Serial No	Lithology	Category of hard rock stratum	Depth of stratum (m)	Distance to Coal Seam 2–3#Top plate distance (m)
Y35	Mudstone	High hard rock stratum	27.6	166.25
Y22	Siltstone	Middle hard rock stratum	14.3	107.86
Y1	Siltstone	Low hard rock stratum	22.26	0.0

### Hard rock energy release calculation

#### Hard rock initial instability energy release law analysis

Following coal mining, the overlying strata may become unstable and shift, eventually reaching a secondary equilibrium state. By analyzing the overlying strata structural features within the stope, it is possible to identify the low, middle, and high hard strata that govern the overlying strata movement and instability.

The calculation method for determining the low, middle, and high hard rock initial instability energy is identical. The hard rock stratum initial instability energy released can expressed as:

15 $$ U_{1} = \frac{{q^{2} }}{450D}\left( {\frac{{a^{5} b^{5} }}{{a^{4} + \frac{8}{49}a^{2} b^{2} + b^{4} }}} \right) $$

where *U*_1_ is the hard rock stratum initial instability energy released (J), D is the hard rock stratum bending stiffness (N m), $D = \frac{{Eh^{3} }}{{12\left( {1 - \mu^{2} } \right)}}$, *h* is the hard rock layer thickness (m), *E* is the hard rock elastic modulus (GPA), *μ* is the hard rock Poisson's ratio, *q* is the vertical uniformly distributed load acting on hard rock stratum, MPa.

The hard rock stratum initial instability energy release depends on various parameters, such as the working face hard rock length (a), the first instability scale (B), the load (Q), the thickness of the rock (h), the elastic modulus (E) and Poisson's ratio (μ). By inputting the relevant hard rock stratum calculation and test results into Formula (15), it is possible to determine the hard rock stratum initial instability energy amount released.

#### Analysis of the periodic instability energy release law for low-level hard rock strata

16 $$ U_{2} = \frac{{a^{5} b^{7} q^{2} }}{{64800\left( {5a^{4} + 20a^{2} b^{2} + 126b^{4} } \right)D}} $$

where *U*_2_ is the low-level hard rock stratum periodic instability energy release (J).

The low-level hard rock stratum periodic instability energy release is determined by various parameters, such as the working face low-level hard rock stratum hanging length (a), periodic instability scale (b), load (q), rock thickness (h), elastic modulus (E) and Poisson's ratio (μ). By inputting the relevant low-level hard rock calculation and test results into Formula (16), it is possible to determine the low-level hard rock periodic instability energy amount released.

By analyzing the periodic instability energy release law for medium- and high-level hard rock, the medium- and high-level hard rock periodic instability energy can be obtained.

17 $$ U_{3} = \frac{{256a^{4} b^{6} q^{2} }}{{6075\left( {336a^{4} + 176a^{2} b^{2} + 165b^{4} } \right)D}} $$

where *U*_3_ is the medium- and high-level hard rock stratum periodic instability energy release (J).

The medium- and high-level hard rock strata periodic instability energy release depends on the medium- and working face high-level hard rock strata hanging length (a), periodic instability scale (B), load (Q), rock thickness (h), elastic modulus (E) and Poisson's ratio (μ). By inputting the relevant medium- and high-level hard rock calculation and test results into Formula (17), it is possible to determine the medium- and high-level hard rock periodic instability energy amount released.

The hard rock structures instability is determined by various parameters, such as the working face hanging length (a), instability scale (b), load (q), rock thickness (h), elastic modulus (E), and Poisson's ratio (μ). The hard rock strata instability energy released is positively correlated with the exposed length (a), instability scale (b), and load (q) in the direction of the working face but negatively correlated with the rock thickness (h), elastic modulus (E), and Poisson's ratio (μ). Hard rock layers with thick and hard characteristics can form a large-scale spatial plate structure, resulting in large hanging length (a) and instability scale (B) along the working face direction, leading to large amounts of energy released during instability.

According to the calculation formula presented in Table 13, the hard rock initial instability energy released is generally greater than that released during periodic instability. Moreover, with the increase in the distance between the hard rock and coal seam roof, the hard rock periodic instability energy released generally increases. Based on research on the instability energy release law of hard rock structures under different conditions, it is necessary to adopt targeted control measures to ensure safe and efficient mining when the hard rock structure instability energy release is significant.

**Table 13:** Hard rock structure initial instability energy release summary.

Space	Stage	
	Initial instability	Periodic instability
Low position Hard rock stratum	$U_{1} = \frac{{q^{2} }}{450D}\left( {\frac{{a^{5} b^{5} }}{{a^{4} + \frac{8}{49}a^{2} b^{2} + b^{4} }}} \right)$	$U_{2} = \frac{{a^{5} b^{7} q^{2} }}{{64800\left( {5a^{4} + 20a^{2} b^{2} + 126b^{4} } \right)D}}$
Middle high position Hard rock stratum		$U_{3} = \frac{{256a^{4} b^{6} q^{2} }}{{6075\left( {336a^{4} + 176a^{2} b^{2} + 165b^{4} } \right)D}}$

### 13,230 working face hard rock layer energy release calculation

The 13,230 working face low and medium hard rock layers instability energy released has been calculated, and the hard rock strata instability energy release law is shown in Table 15.

#### Low-level hard rock instability energy release calculation

① Initial instability energy release calculation
  $$ D = \frac{{Eh^{3} }}{{12\left( {1 - \mu^{2} } \right)}} = {3}.{15} \times {1}0^{13} $$
  $$ U_{1} = \frac{{q^{2} }}{450D}\left( {\frac{{a^{5} b^{5} }}{{a^{4} + \frac{8}{49}a^{2} b^{2} + b^{4} }}} \right) = {334}0{88776}.{7}\;{\text{J}} = {1}.0{5} \times {1}0^{{7}} \;{\text{J}} $$
② Periodic instability energy release calculation
  $$ D = \frac{{Eh^{3} }}{{12\left( {1 - \mu^{2} } \right)}} = {3}.{15} \times {1}0^{{{13}}} $$
  $$ U_{2} = \frac{{a^{5} b^{7} q^{2} }}{{64800\left( {5a^{4} + 20a^{2} b^{2} + 126b^{4} } \right)D}} = {2973}0{69}.{799}\;{\text{J}} = {4}.{51} \times {1}0^{{4}} \;{\text{J}} $$

#### Medium hard rock instability energy release calculation

① Initial instability energy release calculation
  $$ D = \frac{{Eh^{3} }}{{12\left( {1 - \mu^{2} } \right)}} = {1}.{53} \times {1}0^{{{13}}} $$
  $$ U_{1} = \frac{{q^{2} }}{450D}\left( {\frac{{a^{5} b^{5} }}{{a^{4} + \frac{8}{49}a^{2} b^{2} + b^{4} }}} \right) = {7}0{7}0{938}0.{51}\;{\text{J}} = {5}.{42} \times {1}0^{{8}} \;{\text{J}} $$
② Periodic instability energy release calculation
  $$ D = \frac{{Eh^{3} }}{{12\left( {1 - \mu^{2} } \right)}} = {1}.{53} \times {1}0^{{{13}}} $$
  $$ U_{3} = \frac{{256a^{4} b^{6} q^{2} }}{{6075\left( {336a^{4} + 176a^{2} b^{2} + 165b^{4} } \right)D}} = {214541}.{4546}\;{\text{J}} = {7}.{26} \times {1}0^{{6}} \;{\text{J}} $$

Based on research and statistics, the rockburst critical energy in China is generally 10^4^ J^48^. As shown in Table 14, the low- and medium-level hard rock strata initial and periodic instability released energy exceeds this critical energy. When such instability occurs and poses a rockburst risk, appropriate measures should be taken to control the scale of the instability, reduce the energy release, and ensure safe mine production.

**Table 14:** Instability energy release law of hard rock strata in the 13,230 working face.

Horizon	lithology	Category of hard rock stratum	Thickness (m)	Initial instability scale (m)	Initial instability release energy (J)	Periodic instability scale (m)	Periodic instability release energy (J)
Y22 Sandy mudstone	Siltstone	Middle hard rock stratum	14.3	107.81	5.42 × 10^8^	39.3	6.71 × 10^4^
Y1 mudstone	Mudstone	Low hard rock stratum	22.26	73.91	1.05 × 10^7^	25.5	4.51 × 10^4^

### Energy attenuation impact on rockburst and its prevention

During the propagation of energy, the presence of rock mass discontinuities can obstruct stress transmission and propagation. The relationship between the peak velocity and energy is exponentially negative, and this relationship is affected by various coal and rock mass physical and mechanical parameters, such as the elastic modulus, Poisson's ratio, void ratio, friction angle, bulk density, and rock mass cohesion, as well as the propagation distance.

Formula (6) can calculate the energy after attenuation according to the distance between the energy source and the measurement point and the attenuation coefficient. Taking the hard rock stratum overlying the 13,230 working face of the Gengcun Coal Mine as an example, the low hard rock stratum is located directly above the coal seam roof, with a distance of 0 m. Vibration wave energy can directly affect the working face coal body after fracturing. On the other hand, the middle hard rock layer is 93.56 m from the coal seam roof. Using the results calculated by Formula (6), it can be inferred that after the medium hard rock layer instability, the vibration wave energy transmitted to the working face coal body has experienced 53% attenuation, and only 47% of the vibration energy reaches the measurement point.

To prevent rockburst and minimize the energy attenuation effects, it is necessary to take measures to control the structural instability scale and the reduce energy release. One effective approach is to use precutting technology to cut off the rock strata and decrease the rockburst risk.

The energy release results after the initial and periodic instabilities of each layer are as follows:

1. After the low hard rock initial instability, 4.9 × 10^6^ J of energy will act on the coal. After periodic instability, 4.51 × 10^4^ J of energy will act on the coal.
2. After the medium hard rock initial instability, 2.55 × 10^8^ J of energy will act on the coal. After periodic instability, 3.2 × 10^4^ J of energy will act on the coal.

The Gengcun Coal Mine 13,230 working face low and medium strata initial and periodic instabilities increase rockburst risk. To reduce the hard rock structures instability energy released, it is necessary to control the low and medium hard rock structures instability scale and take measures such as roof cutting in advance to reduce rockburst risk.

The corresponding control scale can be determined according to the low- and medium-hard rock strata instability energy released. The coal mine can choose prevention measures based on this, while also considering the actual situation of underground energy monitoring results and rockburst behavior.

### Study on the precutting roof scale of the 13,230 working face

Gengcun Coal Mine 13,230 working face rockburst risk is increased due to the overlying low and middle strata initial and periodic instabilities. To reduce the hard rock stratum structure instability energy released and prevent rockburst, it is suggested to control the low and medium hard rock stratum structure instability scale by combining the mine rockburst critical energy. Precutting can also be applied to further reduce the rockburst risk. According to calculations, the energy released by the initial and periodic instabilities can be controlled at different levels, as shown in Tables 15 and 16, respectively.

**Table 15:** 13,230 working face structure initial instability scale control and released energy reference value.

Stratum position	Hard rock stratum category	Control scale of primary instability of rock strata (m)	Energy released from initial instability of rock strata (J)	Energy from initial instability of rock stratum to measuring point (J)
Y22 sandy mudstone	Medium hard rock stratum	107.81	5.42 × 10^8^	2.55 × 10^8^
		100	3.96 × 10^8^	1.86 × 10^8^
		80	1.46 × 10^8^	6.86 × 10^7^
		70	7.80 × 10^7^	3.66 × 10^7^
		60	3.72 × 10^7^	1.75 × 10^7^
		50	1.52 × 10^7^	7.14 × 10^6^
		40	5.06 × 10^6^	2.38 × 10^6^
		30	1.21 × 10^6^	5.69 × 10^5^
		20	1.60 × 10^5^	7.52 × 10^4^
		18	9.47 × 10^4^	4.45 × 10^4^
		15	3.81 × 10^4^	1.79 × 10^4^
		12	1.25 × 10^4^	5.86 × 10^3^
Y1 mudstone	Low hard rock stratum	73.91	1.05 × 10^7^	4.94 × 10^6^
		60	3.78 × 10^6^	1.78 × 10^6^
		50	1.53 × 10^6^	7.19 × 10^5^
		40	5.06 × 10^5^	2.38 × 10^5^
		30	1.21 × 10^5^	5.69 × 10^4^
		20	1.59 × 10^4^	7.47 × 10^3^

**Table 16:** 13,230 working face structure periodic instability scale control and released energy reference value.

Stratum position	Hard rock stratum category	Control scale of periodic instability of rock strata (m)	Release energy of periodic instability of rock strata (J)	Energy from period instability of rock stratum to measuring point (J)
Y22 sandy mudstone	Medium hard rock stratum	39.3	6.71 × 10^4^	3.15 × 10^4^
		35	3.38 × 10^4^	1.59 × 10^4^
		30	1.35 × 10^4^	6.35 × 10^3^
Y1 mudstone	Low hard rock stratum	25.5	4.51 × 10^4^	2.12 × 10^4^
		20	8.48 × 10^3^	3.99 × 10^3^

Table 15 outlines the control and release energy values of the 13,230 working face structure initial instability scale. For instance, when the roof strata initial instability energy released is controlled below 10^6^ J, the medium hard rock strata can be precut at 30 m (considering for attenuation), and the low hard rock stratum can be precut at 50 m. Similarly, when the roof strata initial instability energy released is controlled below 10^5^ J and below 10^4^ J, different precutting distances for medium and low hard rock strata can be determined.

Table 16 illustrates the 13,230 working face structural periodic instability scale control and energy release reference values. For example, when the roof strata periodic instability energy released is controlled below 10^6^ J, medium hard rock stratum and the low hard rock stratum precutting may not be necessary. However, when the roof strata periodic instability energy released is controlled below 10^5^ J and below 10^4^ J, medium and low hard rock strata precutting distances can be determined.

In China, the critical rockburst energy is 10^4^ J to 10^6^ J. The following control scales are recommended:

1. The initial instability release energy should be controlled below 10^6^ J. The middle hard rock can be cut off in advance at 30 m (considering attenuation), and the roof of low hard rock can be cut in advance at 50 m.
2. The initial instability release energy should be controlled below 10^5^ J. The middle hard rock can be cut off in advance at 20 m, and the low hard rock roof can be cut in advance at 30 m.
3. The initial instability release energy should be controlled below 10^4^ J. The middle hard rock can be cut off in advance at 12 m, and the low hard rock roof can be cut in advance at 20 m.
4. The periodic instability release energy should be controlled below 10^6^ J, and medium hard rock and low hard rock roof cutting in advance is not required.
5. The periodic instability release energy should be controlled below 10^5^ J. The medium hard rock layer roof can be cut in advance at an interval of 39.3 m, and the low hard rock roof can be cut in advance at 25.5 m.
6. The periodic instability release energy should be controlled below 10^4^ J. The medium hard rock layer roof can be cut in advance at an interval of 30 m, and the low hard rock roof can be cut in advance at 20 m.

Refer to Table 17 for the 13,230 working face first and periodic instability precutting scale reference value.

**Table 17:** 13,230 working face initial and periodic stability cutting scale in advance reference value.

Horizon	Hard rock stratum category	Energy after control (J)	Top cutting scale of initial instability (m)	Scales of periodic instability cutoff (m)
Y22 Medium coarse sandstone	Median hardness rock stratum	10^6^	50	–
		10^5^	30	–
		10^4^	20	39.3
		10^3^	12	30
Y1 Siltstone	Hard low position rock stratum	10^6^	73.91	–
		10^5^	50	–
		10^4^	30	25.5

## Conclusions

1. Based on the relevant mechanical knowledge, a blasting model has been established that shows energy will diffuse around the coal and rock mass after a rockburst occurs according to a certain law. Different mechanical parameters will have varying effects on energy attenuation. Through numerical simulation using Midas GTX, the different mechanical parameter weights were determined. Specifically, Poisson's ratio weight is 0.3838, elastic modulus weight is 0.2558, void ratio weight is 0.1719, the weight of the unit weight is 0.0963, friction angle weight is 0.0580, and cohesion weight is 0.0342. By using these weights, an equation has been derived for calculating the coal and rock mass attenuation coefficient of energy.
  $$ K = \frac{{2.07 \times 10^{ - 3} k_{1} + 2.61 \times 10^{ - 3} k_{2} + 3.06 \times 10^{ - 4} k_{3} + 5.22 \times 10^{ - 5} k_{4} + 7.03 \times 10^{ - 5} k_{5} + 2.77 \times 10^{ - 5} k_{6} }}{0.3838 + 0.2558 + 0.1719 + 0.0963 + 0.0580 + 0.0342} $$
2. To determine the concrete test bench attenuation coefficient of energy, we established a test setup consisting of a 1 m × 1 m × 1 m structure. By varying the height and mass (100 g, 200 g, 300 g) of the falling ball, as well as the distance between the force point and the sensor, we were able to obtain the necessary data. Using the energy attenuation formula, we calculated an attenuation coefficient of 0.00455. This indicates that energy attenuates at a rate of 0.455% per unit distance. Additionally, we found that the energy attenuation amplitude was 26%, meaning that only 26% of the initial energy was present at the measurement point. The remaining 74% of the energy was in the form of vibration energy acting on the measuring point.
3. We conducted a mechanical parameter test on the cement test model and calculated the proportion coefficients between the test model and the basic model. Specifically, we found that k_1_ was 1.4, k_2_ was 1.04, k_3_ was 1, k_4_ was 1.58, k_5_ was 1, and k_6_ was 1.2. By substituting these proportion coefficients into the attenuation coefficient formula, we calculated an attenuation coefficient of k = 0.00607. This is on the same order of magnitude as the previous value of 0.00455 obtained from the previous test and therefore confirms our earlier findings. The test verification is consistent, and we can conclude that the mechanical parameter test results are reliable.
4. Based on the energy attenuation range, we have developed a 13,230 working face roof cutting guide. Our guide includes middle and low hard rock layers specific cutting distances, as well as guidelines for controlling the instability release energy. For initial instability release energy controlled below 10^6^ J, we recommend middle hard rock layer roof cutting in advance at 50 m and the low hard rock layer in advance at 73.91 m. For initial instability release energy controlled below 10^5^ J, we recommend medium hard rock layer roof cutting in advance at 30 m and the low hard rock layer roof in advance at 50 m. For initial instability release energy controlled below 10^4^ J, we recommend medium hard rock layer roof cutting in advance at 20 m and the low hard rock layer roof in advance at 30 m. For periodic instability release energy controlled below 10^6^ J, medium and low hard rock layer roof advance cutting is not required. For periodic instability release energy controlled below 10^5^ J, medium and low hard rock layer roof advance cutting is not required. For periodic instability release energy controlled below 10^4^ J, we recommend medium hard rock layer roof advance cutting at an interval of 39.3 m and low hard rock layer roof advance cutting at 25.5 m. Following these guidelines will help to ensure the safety and stability of the working face during roof cutting operations.

## Data Availability

The data used to support the findings of this study are available from the corresponding author upon request.
